# Supplementary material for: Integrated histopathology of the human pancreas throughout stages of type 1 diabetes progression
Source: Nat Commun. 2026 Feb 11;17:4293. doi: 10.1038/s41467-026-68610-1 (PMC13168456; doi:10.1038/s41467-026-68610-1)
Supplement: Supplementary file 1 — Supplementary Information [file 41467_2026_68610_MOESM1_ESM.pdf]

Integrated histopathology of the human pancreas throughout stages of type 1 diabetes progression

Verena van der Heide, Sara McArdle, Michael S. Nelson, Karen Cerosaletti, Sacha Gnjatic, Zbigniew Mikulski, Amanda L. Posgai, Irina Kusmartseva, Mark A. Atkinson & Dirk Homann

SUPPLEMENTARY FIGURES & TABLES

Supplementary Figures S1 – S8. ***Figs.S1 – S8*** feature additional data related to ***Figs.1 – 8***.

Supplementary Table S1. ***Table S1*** features information about antibodies and MICSSS staining conditions.

Additional Supplementary Data (Supplementary Data 1: detailed information about pancreas specimens, demographic and clinical donor metadata, and HLA haplotype T1D risk; Supplementary Data 2: summary of properties of donor tissue sections and of all ~25,000 individual islets captured in the present study) are provided as separate documents.

Figure S1

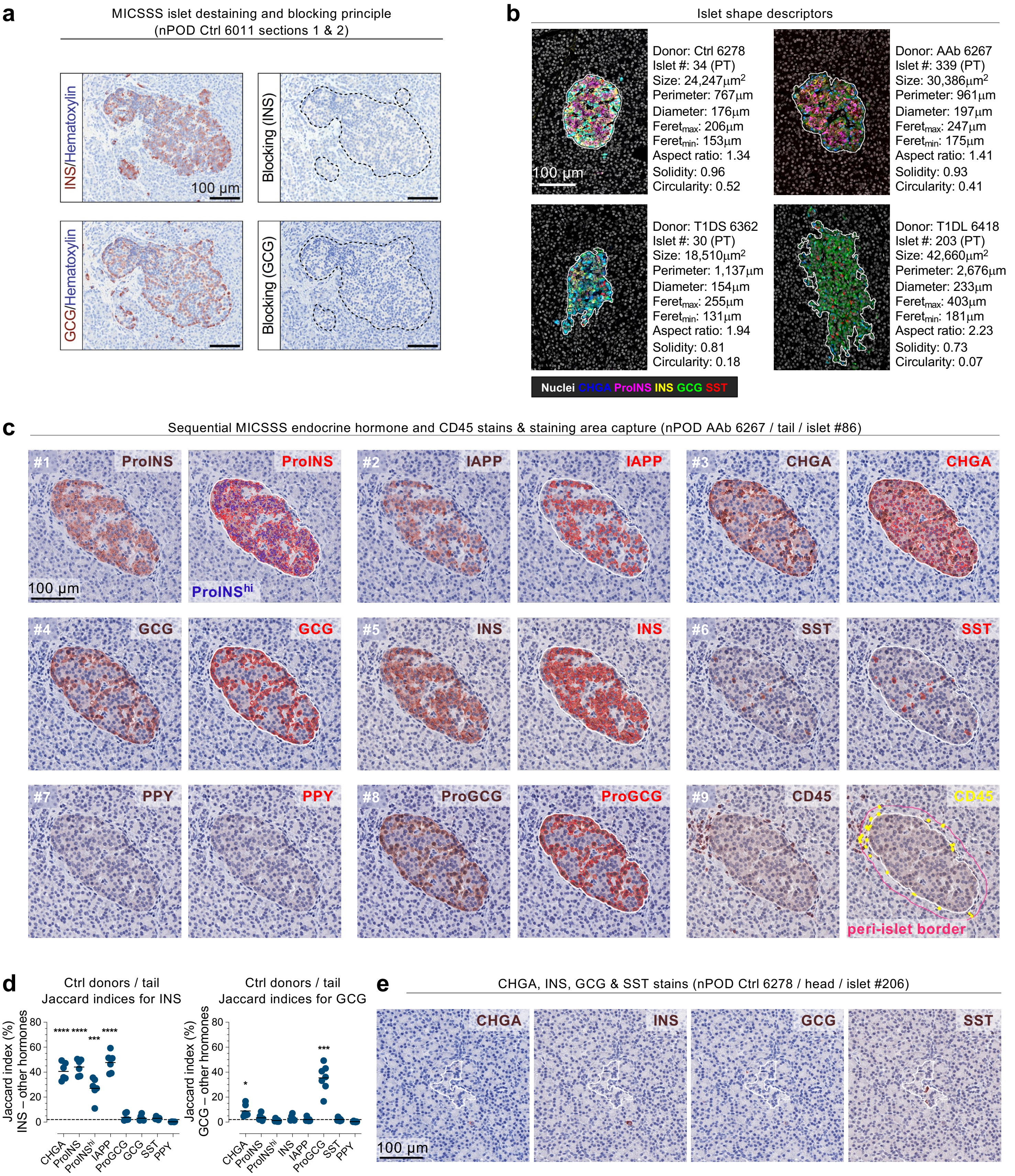
a
MICSSS islet destaining and blocking principle
(nPOD Ctrl 6011 sections 1 & 2)
INS/Hematoxylin
Blocking (INS)
GCG/Hematoxylin
Blocking (GCG)
100 µm
b
Islet shape descriptors
Donor: Ctrl 6278
Islet #: 34 (PT)
Size: 24,247µm2
Perimeter: 767µm
Diameter: 176µm
Feretmax: 206µm
Feretmin: 153µm
Aspect ratio: 1.34
Solidity: 0.96
Circularity: 0.52
Donor: AAb 6267
Islet #: 339 (PT)
Size: 30,386µm2
Perimeter: 961µm
Diameter: 197µm
Feretmax: 247µm
Feretmin: 175µm
Aspect ratio: 1.41
Solidity: 0.93
Circularity: 0.41
Donor: T1DS 6362
Islet #: 30 (PT)
Size: 18,510µm2
Perimeter: 1,137µm
Diameter: 154µm
Feretmax: 255µm
Feretmin: 131µm
Aspect ratio: 1.94
Solidity: 0.81
Circularity: 0.18
Donor: T1DL 6418
Islet #: 203 (PT)
Size: 42,660µm2
Perimeter: 2,676µm
Diameter: 233µm
Feretmax: 403µm
Feretmin: 181µm
Aspect ratio: 2.23
Solidity: 0.73
Circularity: 0.07
100 µm
Nuclei CHGA ProINS INS GCG SST
c
Sequential MICSSS endocrine hormone and CD45 stains & staining area capture (nPOD AAb 6267 / tail / islet #86)
#1 ProINS
ProINS
ProINShi
100 µm
#2 IAPP
IAPP
#3 CHGA
CHGA
#4 GCG
GCG
#5 INS
INS
#6 SST
SST
#7 PPY
PPY
#8 ProGCG
ProGCG
#9 CD45
CD45
peri-islet border
d
Ctrl donors / tail
Jaccard indices for INS
Jaccard index (%)
INS – other hormones
Ctrl donors / tail
Jaccard indices for GCG
Jaccard index (%)
GCG – other hromones
CHGA
ProINS
ProINShi
IAPP
ProGCG
GCG
SST
PPY
INS
e
CHGA, INS, GCG & SST stains (nPOD Ctrl 6278 / head / islet #206)
CHGA
INS
GCG
SST
100 µm

Supplementary Figure S1. MICSSS staining and semi-automated image analysis of pancreatic tissue sections. a., MICSSS destaining/blocking illustrated for INS and GCG stains on adjacent tissue slides. **b.,** pseudofluorescent rendering of four islets (islet objects generated as detailed in ***Fig.1d*** and Methods) and corresponding islet shape descriptors. **c.**, sequential MICSSS staining of eight pancreatic endocrine hormones and CD45. Each image pair features the native IHC image (left, number in upper left corner indicates position in staining sequence) and the same image overlaid with automatically captured staining area traces (right) and islet perimeter (white) (note that ProINS images are duplicated here from ***Fig.1f***, and islet #86 does not contain PPY^+ gamma cells). Automated $CD45^+$ cell captures (highlighted in yellow) are restricted to areas within the peri-islet border (pink; set at a distance of 20 μm from islet perimeter) since classifier training did not include immune cells located outside that border. **d.,** to assess the relative extent of staining overlap between INS or GCG and other endocrine hormones, respective Jaccard indices were determined for Ctrl donor PT sections by dividing the size of the staining area intersection by the size of the union of staining areas (expressed as fraction [%] of staining area) for each combination of hormone staining pairs; scatter plots display individual donor means (blue circles) and group means (black horizontal bars); INS and CHGA data from Ctrl 6162 were excluded due to notably weak staining; asterisks indicate statistical significance (*$p<0.05$, **$p<0.01$, ***$p<0.001$ and ****$p<0.0001$) in two-tailed one sample t tests for values >2% (dashed line) staining overlap. **e.,** CHGA, INS, GCG and SST brightfield stains (brown) of PPY^+ islet #206 (*cf.,* ***Fig.1h***); note the absence of alpha and beta cells as well as little CHGA expression.

Figure S2

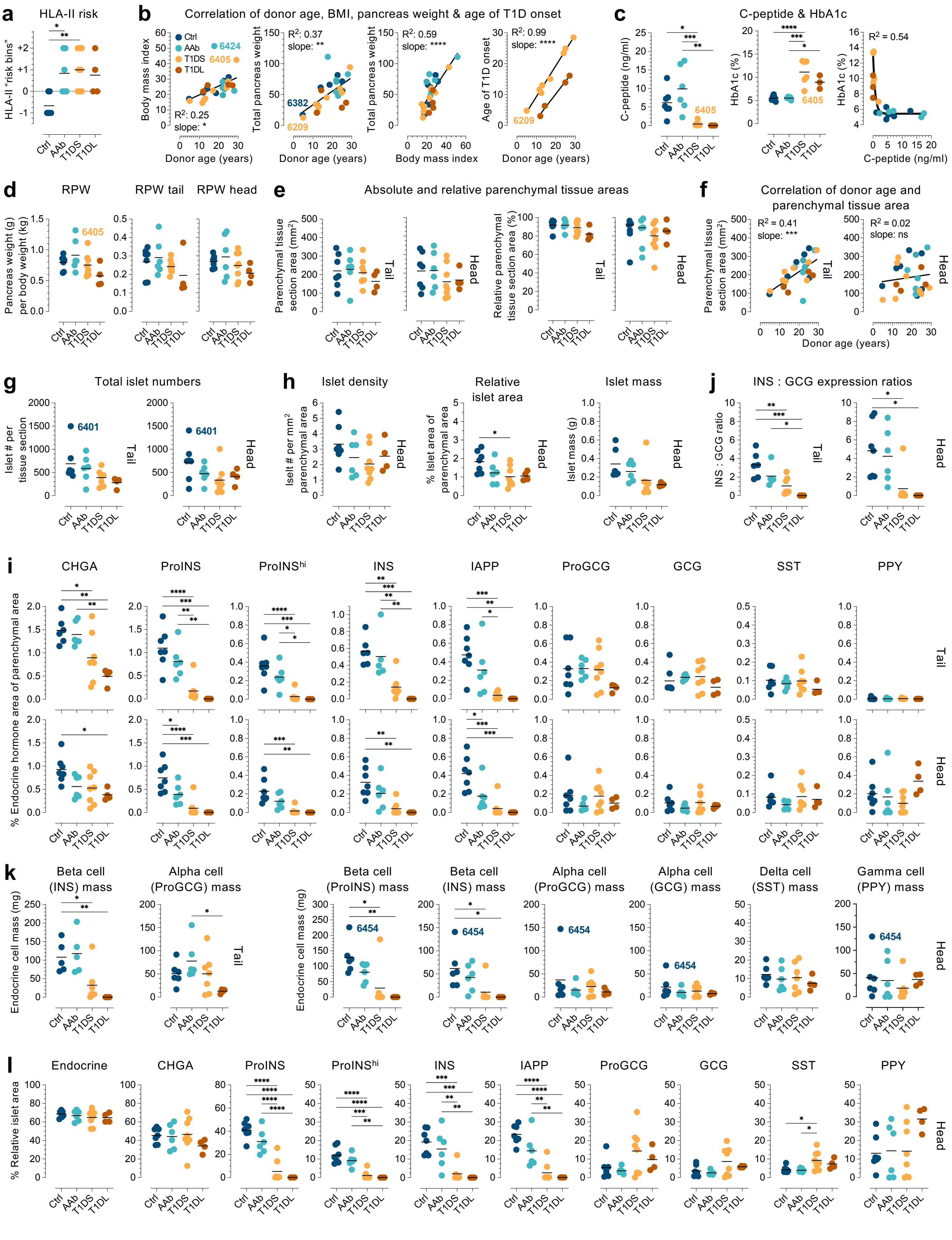

a
HLA-II risk
HLA-II "risk bins"
b
Correlation of donor age, BMI, pancreas weight & age of T1D onset
Ctrl
AAb
T1DS
T1DL
6424
6405
6382
6209
Body mass index
Total pancreas weight
Age of T1D onset
Donor age (years)
R2: 0.25
slope: *
R2: 0.37
slope: **
R2: 0.59
slope: ****
R2: 0.99
slope: ****
c
C-peptide & HbA1c
C-peptide (ng/ml)
HbA1c (%)
R2 = 0.54
d
RPW
RPW tail
RPW head
Pancreas weight (g) per body weight (kg)
e
Absolute and relative parenchymal tissue areas
Parenchymal tissue section area (mm2)
Relative parenchymal tissue section area (%)
Tail
Head
f
Correlation of donor age and parenchymal tissue area
R2 = 0.41
slope: ***
R2 = 0.02
slope: ns
g
Total islet numbers
Islet # per tissue section
6401
h
Islet density
Relative islet area
Islet mass
Islet # per mm2 parenchymal area
% Islet area of parenchymal area
Islet mass (g)
j
INS : GCG expression ratios
INS : GCG ratio
i
CHGA
ProINS
ProINShi
INS
IAPP
ProGCG
GCG
SST
PPY
% Endocrine hormone area of parenchymal area
k
Beta cell (INS) mass
Alpha cell (ProGCG) mass
Beta cell (ProINS) mass
Alpha cell (GCG) mass
Delta cell (SST) mass
Gamma cell (PPY) mass
Endocrine cell mass (mg)
6454
l
Endocrine
% Relative islet area

Supplementary Figure S2. Pancreatic donor, tissue and islet properties across stages of T1D progression. Unless noted otherwise, scatter plots display individual donor means (colored circles) and group means (black horizontal bars). **a.,** HLA-II haplotypes were "binned" according to ref.[1] and assigned the following numerical "risk values": protective = -1, neutral = 0, moderate risk = +1, high risk = +2. **b. & c.,** correlation of donor age, BMI, pancreas weight and age of T1D onset as well as C-peptide and HbA1c. Goodness of linear or exponential curve fit (R^2) is indicated, and asterisks indicate statistical significance of slope deviation from zero. "Outliers" as based on severe obesity (AAb 6424, T1DS 6405), very young age (Ctrl 6382, T1DS 6209) and comparatively better glucose handling (T1DS 6405) are indicated (HbA1c values for Ctrl 6162, AAb 6310, T1DS 6209, T1DS 6247 and T1DL 6180 were not available). **d.,** relative pancreatic weight (RPW: pancreas weight [g] / body weight [kg]) was calculated for the entire organ (left) as well as PT (middle) and PH (right) regions. **e. & f.,** absolute and relative parenchymal tissue section areas, and correlation of former with donor age in PT but not PH (significance of slope deviation from zero for simple linear regression is indicated; also, note that the respective correlation patterns are reinforced when analyses are restricted to only those donors from whom complete pancreatic tissue cross-sections were obtained [PT: R^2=0.53, slope p=0.0002; PH: R^2=0.04, slope p=ns], *cf.,* ***Supplementary Data 1*** and standard nPOD pancreas processing protocol in ref.[2]). **g. & h.,** absolute numbers of islets in PT and PH tissue sections as well as islet densities (numbers of islets per mm^2 parenchymal tissue area), cumulative islet areas and islet mass in PH. **i.,** fraction of indicated total endocrine hormone staining areas normalized to parenchymal tissue areas in the PT (top) and PH (bottom). A reduction of ProINS areas in AAb donors would appear to contradict a reported elevation of total ProINS expression and ProINS:INS ratios in AAb *vs.* Ctrl donors[3]. However, those outcomes are in part dependent on the capture of small endocrine clusters (<1,000 μm^2) excluded in the present analyses; they appear to be more pronounced in single than double AAb^+ donors[3]; and ProINS:INS expression ratios become exceedingly variable with disease advancement[3-5]. Thus, our observations do not challenge the emerging paradigm of altered beta cell prohormone processing in T1D progression[6]. **j.,** INS:GCG expression ratios were calculated with individual donor values featured in panel i. **k.,** endocrine cell type mass was calculated with respective PT and PH data in panel i and corresponding PT or PH weights (***Supplementary Data 1***) under exclusion of the two 5-year-old donors Ctrl 6382 and T1DS 6209; Ctrl case 6405 is indicated due to their high alpha and beta cell mass. **l.,** fraction of indicated relative endocrine hormone staining areas in individual islets of the PH ("endocrine" refers to the union of all hormone staining areas). Due to notably weak CHGA and/or INS staining of PT but not PH sections from two donors (CHGA: Ctrl 6162; INS: Ctrl 6162, AAb 6450), the respective PT data are excluded in panels i-k. Scatter plots display individual donor means (colored circles) and, where applicable, group means (black horizontal bars) with n= 7 Ctrl, 6 AAb, 8 T1DS and 4 T1DL donors. Statistical analyses were conducted with ordinary one-way ANOVA and Tukey's multiple comparisons test adhering to the following convention: *p<0.05, **p<0.01, ***p<0.001, ****p<0.0001; ns, non-significant.

Figure S3

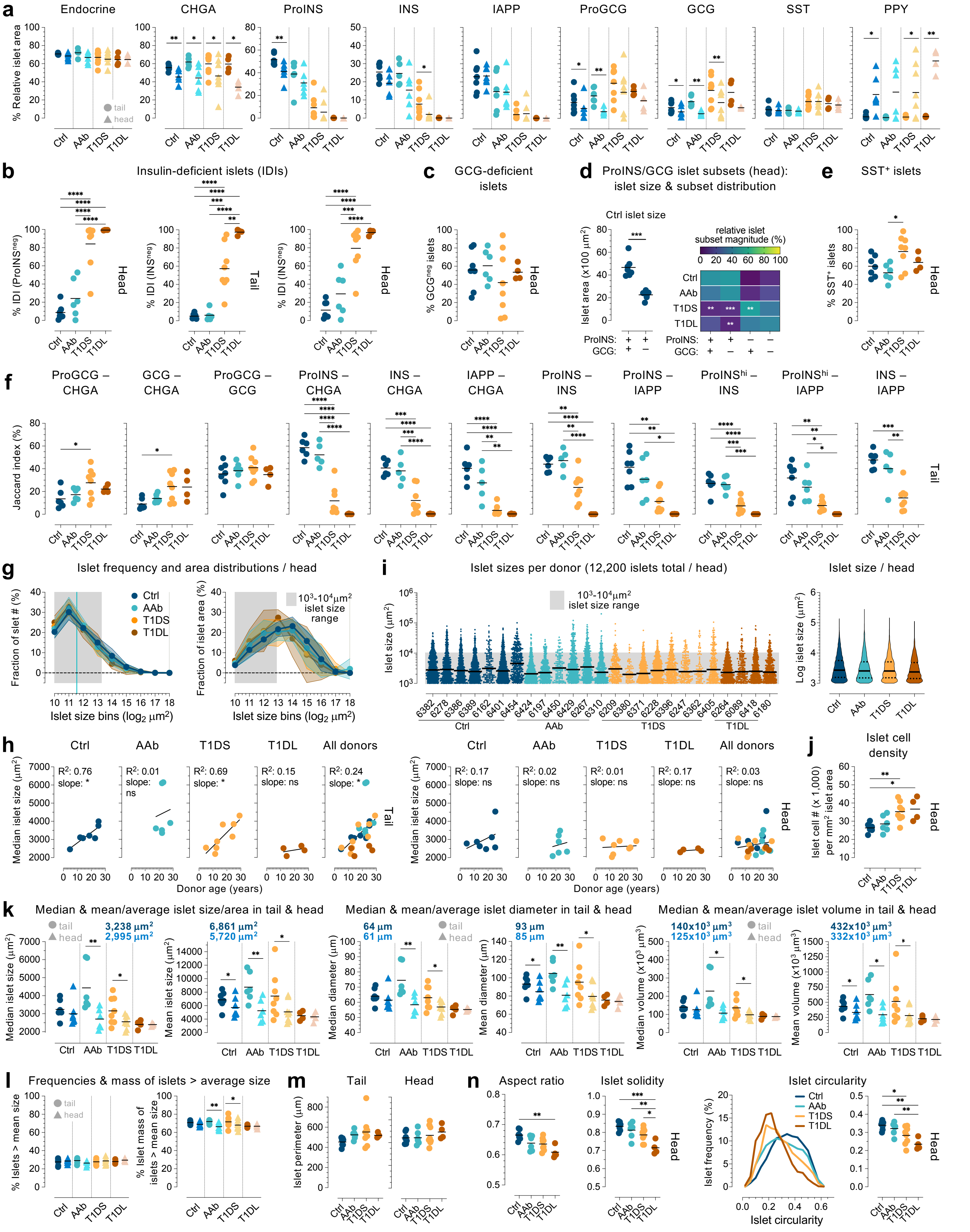

a
Endocrine
CHGA
ProINS
INS
IAPP
ProGCG
GCG
SST
PPY
% Relative islet area
tail
head
Ctrl
AAb
T1DS
T1DL
b
Insulin-deficient islets (IDIs)
% IDI (ProINSneg)
% IDI (INSneg)
Head
Tail
c
GCG-deficient islets
% GCGneg islets
d
ProINS/GCG islet subsets (head): islet size & subset distribution
Ctrl islet size
Islet area (x100 µm2)
relative islet subset magnitude (%)
ProINS:
GCG:
e
SST+ islets
% SST+ islets
f
ProGCG – CHGA
GCG – CHGA
ProGCG – GCG
ProINS – CHGA
INS – CHGA
IAPP – CHGA
ProINS – INS
ProINS – IAPP
ProINShi – INS
ProINShi – IAPP
INS – IAPP
Jaccard index (%)
g
Islet frequency and area distributions / head
Fraction of islet # (%)
Fraction of islet area (%)
Islet size bins (log2 µm2)
103-104µm2 islet size range
i
Islet sizes per donor (12,200 islets total / head)
Islet size (µm2)
Islet size / head
Log islet size (µm2)
6382 6278 6386 6389 6162 6401 6454 6424 6197 6450 6429 6267 6310 6209 6380 6371 6228 6396 6247 6362 6405 6264 6089 6418 6180
h
All donors
Median islet size (µm2)
Donor age (years)
R2: 0.76 slope: *
R2: 0.01 slope: ns
R2: 0.69 slope: *
R2: 0.15 slope: ns
R2: 0.24 slope: *
R2: 0.17 slope: ns
R2: 0.02 slope: ns
R2: 0.01 slope: ns
R2: 0.17 slope: ns
R2: 0.03 slope: ns
j
Islet cell density
Islet cell # (x 1,000) per mm2 islet area
k
Median & mean/average islet size/area in tail & head
Median & mean/average islet diameter in tail & head
Median & mean/average islet volume in tail & head
3,238 µm2
2,995 µm2
6,861 µm2
5,720 µm2
64 µm
61 µm
93 µm
85 µm
140x103 µm3
125x103 µm3
432x103 µm3
332x103 µm3
Median islet size (µm2)
Mean islet size (µm2)
Median diameter (µm)
Mean diameter (µm)
Median volume (x103 µm3)
Mean volume (x103 µm3)
l
Frequencies & mass of islets > average size
% Islets > mean size
% Islet mass of islets > mean size
m
Tail
Head
Islet perimeter (µm)
n
Aspect ratio
Islet solidity
Islet circularity
Islet frequency (%)

Supplementary Figure S3. Properties of islets and islet subsets across stages of T1D progression. a., for comparative purposes, panel a features the same data as in ***Figs.1k & S2l*** to contrast relative islet hormone staining areas in PT *vs.* PH. **b.,** frequencies of IDIs with <1% ProINS or <0.1% INS expression. **c.,** frequencies of islets with <1% GCG expression. **d.,** left: size of indicated PH islet subsets (individual donor medians and collective means); right: heatmap displaying the relative magnitude (donor group means) of indicated ProINS/GCG PH islet subsets in each T1D stage (asterisks pertain to relative abundance of each islet subset in comparison to Ctrl donors)**. e.,** frequencies of islets with ≥1% SST expression. **f.,** Jaccard indices were calculated as detailed in Methods for dual combinations of hormones co-expressed by alpha cells (CHGA, ProGCG, GCG) or beta cells (CHGA, ProINS, $ProINS^{hi}$, INS, IAPP) and are displayed as a function of T1D stage. The reduction of ProINS-INS Jaccard indices with disease progression appears to contravene the reported increase of ProINS/INS co-localization in T1DS donors. However, the latter phenomenon was largely restricted to young children with a more aggressive "endotype 1" T1D version[7], and the Manders overlap coefficient (MOC) used for quantification of ProINS/INS co-localization[7] is contingent on confocal microscopy and not immediately comparable to the Jaccard index. Nevertheless, the concurrently reported decline of the ProINS/INS MOC in T1D *vs.* Ctrl subjects ≥13 years of age[7] readily aligns with the reduction of ProINS-INS Jaccard indices as shown here for T1DS and T1DL cohorts. **g.**, islet frequencies (left) and respective contributions to overall islet area (right) of $\log_2$-transformed, bin-stratified islet sizes in the PH (data are mean±95% CI displayed as error bands; vertical dashed line in left panel set at 3,000 μm^2). **h.,** correlation of median islet size with donor age in PT and PH (goodness of linear regression fit and significance of slope deviation from zero are indicated). While AAb donors are ~4 years older than other donors (*cf.,* ***Supplementary Data 1***), the ~37% islet size increase in the PT of AAb *vs.* Ctrl donors (***Fig.2j***) exceeds an estimated age-associated islet size gain of ~17% as shown here. **i.,** left: distribution of islet sizes in the PH comprising all 12,200 individual islets stratified according to donor group and individual donors therein (within each donor group, donors are ordered according to increasing age; black bars: medians). Right: the violin plot summarizes donor group-specific islet size distributions (median/quartiles indicated) with statistical differences calculated using a mixed model (no significant differences recorded). **j.,** islet cell densities (geometric means of islet cell numbers per mm^2 islet area). **k.,** islet sizes, diameters and volumes in PT *vs.* PH stratified according to T1D stage. To facilitate comparisons with the published literature, the panels feature both median and mean islet areas as determined from direct islet area measurements as well as median and mean islet diameters and volumes calculated from islet areas under the assumption of circularity = 1 and sphericity = 1 (*cf.,* equations in ***Fig.1e***; although these assumptions constitute an oversimplification they are in line with similar calculations made in other reports[8]). The values in the upper left panel corners indicate median or mean values for Ctrl donor islets in PT (dark blue) and PH (lighter blue) (the difference between median Ctrl PT *vs.* PH islet sizes, diameters and volumes remains non-significant even after outlier removal from the PH group). **l.,** frequencies and relative mass contributions of islets greater than average size in PT *vs.* PH stratified according to T1D stage. **m.,** average islet perimeters in PT and PH. **n.,** islet aspect ratio ($Feret_{min}$ / $Feret_{max}$ diameters), solidity (area / convex area) and circularity (4π x area / $perimeter^2$) in the PH; the binned histograms display donor group-specific islet circularity distributions. Due to notably weak CHGA and/or INS staining of PT but not PH sections from two donors (CHGA: Ctrl 6162; INS: Ctrl 6162, AAb 6450), the respective PT data are excluded in panels a, b and f. Unless noted otherwise, scatter plots display individual donor means (colored symbols) and group means (black horizontal bars) with n= 7 Ctrl, 6 AAb, 8 T1DS and 4 T1DL donors. Statistical analyses were conducted with two-tailed Student's t-test (panels a, d/left, k, l), ordinary one-way ANOVA and Tukey's multiple comparisons test (panels b, c, d/right, e, f, j, m, n), "bin-specific" one-way ANOVA (panel g), or mixed model analyses (panel i/right, see Methods); both t-tests and ANOVAs adopt the following convention: *p<0.05, **p<0.01, ***p<0.001, ****p<0.0001.

Figure S4

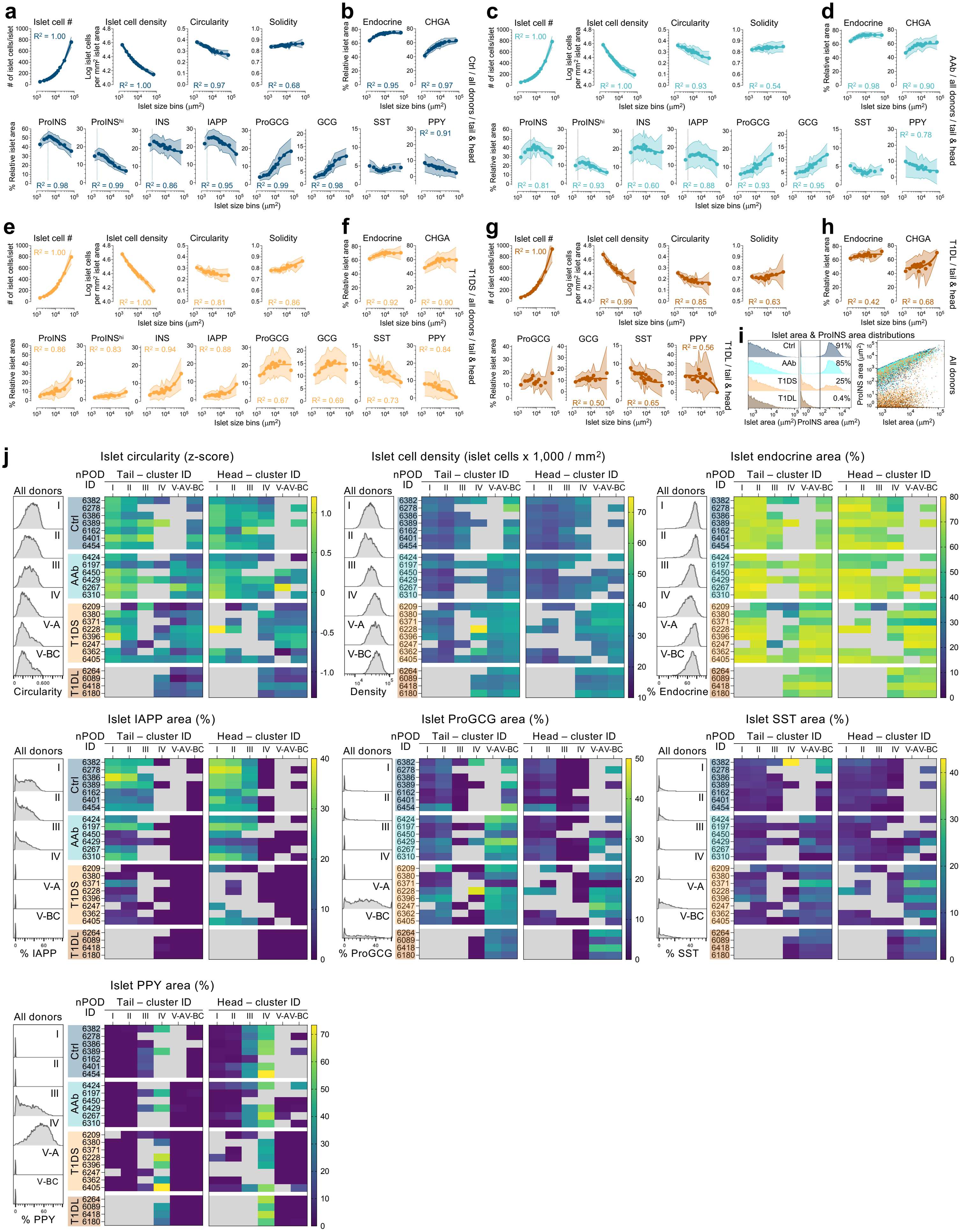

a
Islet cell #
Islet cell density
Circularity
Solidity
# of islet cells/islet
Log islet cells per mm² islet area
Islet size bins (μm²)
b
Endocrine
CHGA
% Relative islet area
Ctrl / all donors / tail & head
c
d
AAb / all donors / tail & head
ProINS
ProINShi
INS
IAPP
ProGCG
GCG
SST
PPY
e
f
T1DS / all donors / tail & head
g
h
T1DL / tail & head
i
Islet area & ProINS area distributions
Ctrl
AAb
T1DS
T1DL
91%
85%
25%
0.4%
Islet area (μm²)
ProINS area (μm²)
ProINS area (μm²)
All donors
j
Islet circularity (z-score)
Islet cell density (islet cells x 1,000 / mm²)
Islet endocrine area (%)
Islet IAPP area (%)
Islet ProGCG area (%)
Islet SST area (%)
Islet PPY area (%)
nPOD ID
Tail – cluster ID
Head – cluster ID
I
II
III
IV
V-A
V-BC
All donors
Ctrl
AAb
T1DS
T1DL
6382
6278
6386
6389
6162
6401
6454
6424
6197
6450
6429
6267
6310
6209
6380
6371
6228
6396
6247
6362
6405
6264
6089
6418
6180
Circularity
Density
% Endocrine
% IAPP
% ProGCG
% SST
% PPY

Supplementary Figure S4. Correlations of islet size with islet properties & islet properties stratified according to UMAP cluster affiliation. a.-h., combined PT and PH islets ranging in size from 10^3 - 10^5μm^2 from individual donors within indicated T1D stage groups were allocated to 14 bins (variable islet size bins to accommodate islet property analyses with sufficient numbers of islets in each bin; 1,000-10,000 μm^2: 9 bins, 10,000-15,000 μm^2: 2 bins, 15,000-20,000 μm^2: 1 bin, 20,000-40,000 μm^2: 1 bin, 40,000-100,000 μm^2: 1 bin), and respective bin centers are plotted against corresponding islet features (islet cell numbers, density, circularity, solidity and relative hormone staining areas). Data are mean±95% CI displayed as error bands, and in cases with <3 islets/bin, corresponding values were excluded. All curve fits describe exponential relations (one-phase association or decay; two-phase exponential decay for log-transformed islet cell numbers) and goodness of fit (R^2) is noted where applicable (curve fits with $R^2 < 0.4$ were omitted); in some cases, curve fitting was performed under exclusion of data points for very small islets as indicated by vertical dotted lines in respective plots. Note that the data for islet cell density, circularity, solidity, endocrine and CHGA as well as Ctrl, AAb and T1DS ProINS, INS, IAPP, GCG and SST contents are the same as in ***Fig.2n-q*** and shown here individually for clarification of error distributions and curve fits. Panels a & b, Ctrl donors; c & d, AAb donors; e & f, T1DS donors; and g & h, T1DL donors (note that while an overall contingency of islet properties on islet size remains applicable to the T1DL stage, substantially increased variability among individual donors and islets results in a deterioration of these associations - in particular for ProGCG, GCG and PPY - reflecting a general disorder of islet composition). **i.,** left: histograms of islet size and ProINS area size distribution stratified according to T1D donor group; the vertical bar in ProINS histograms is set at 100 μm^2 corresponding to ~1 endocrine cell, and values are percentages of islets with ProINS staining areas of ≥100 μm^2. Right: dot plot displaying islet area *vs.* ProINS area for individual islets color-coded according to T1D stage group. **j.,** histograms display cluster-stratified frequency distributions of combined PT and PH islets from all donor groups (Ctrl, AAb, T1DS, T1DL) according to indicated modalities (islet circularity and cellular density as well as relative endocrine and hormone staining areas for IAPP, ProGCG, SST, PPY); the adjacent heatmaps stratify the same parameters across T1D stage, individual donors (listed in order of increasing age within each group), islet cluster affiliation, and PT/PH regions. Note that not all donors have islets populating each cluster and we further omitted values if clusters contained <3 islets or <2 donors; missing and excluded values rendered in gray.

Figure S5a/b

Pancreatic tail – islet properties across UMAP clusters grouped according to T1D stage

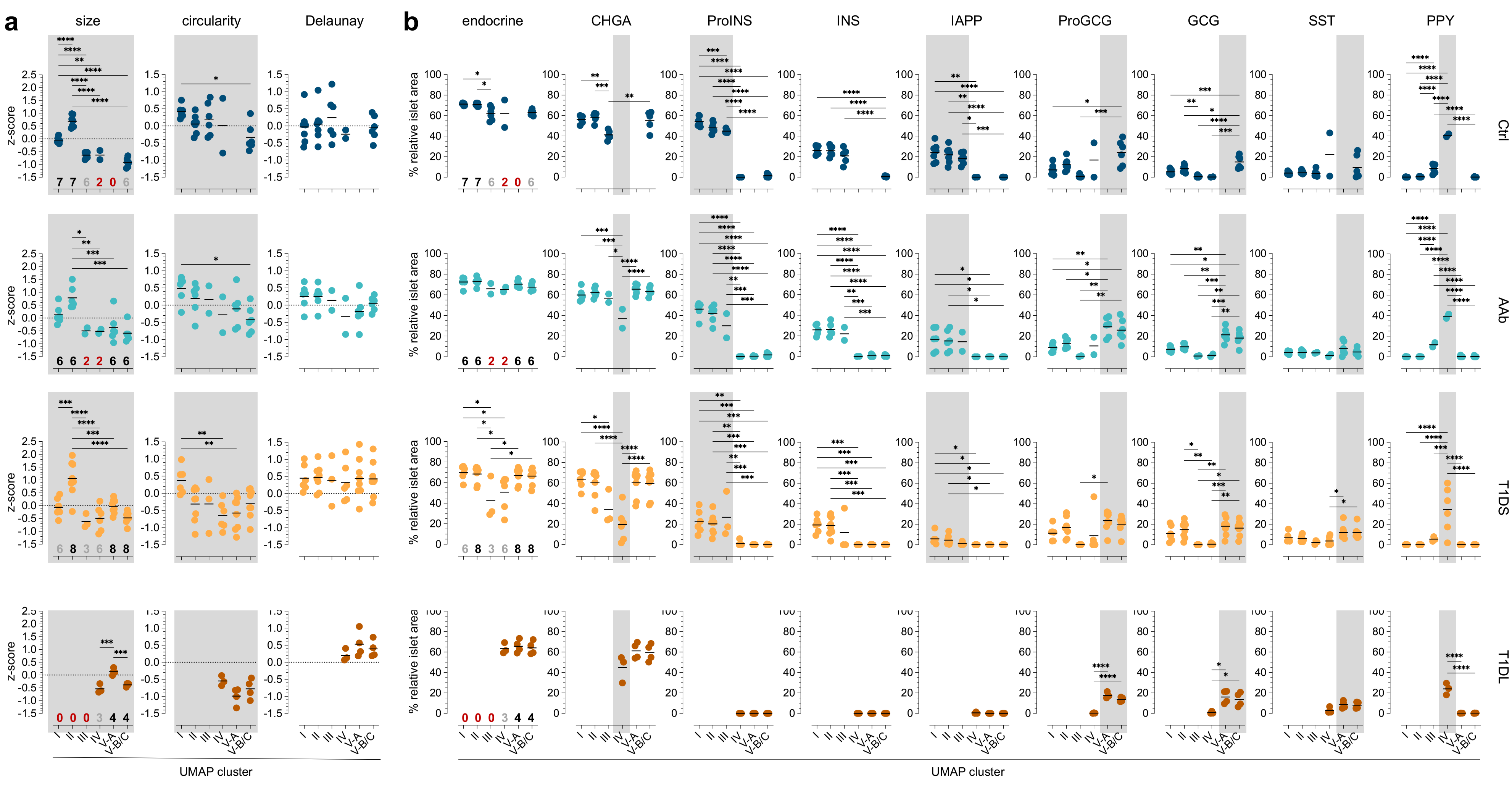

Figure S5a/b

Figure S5c/d

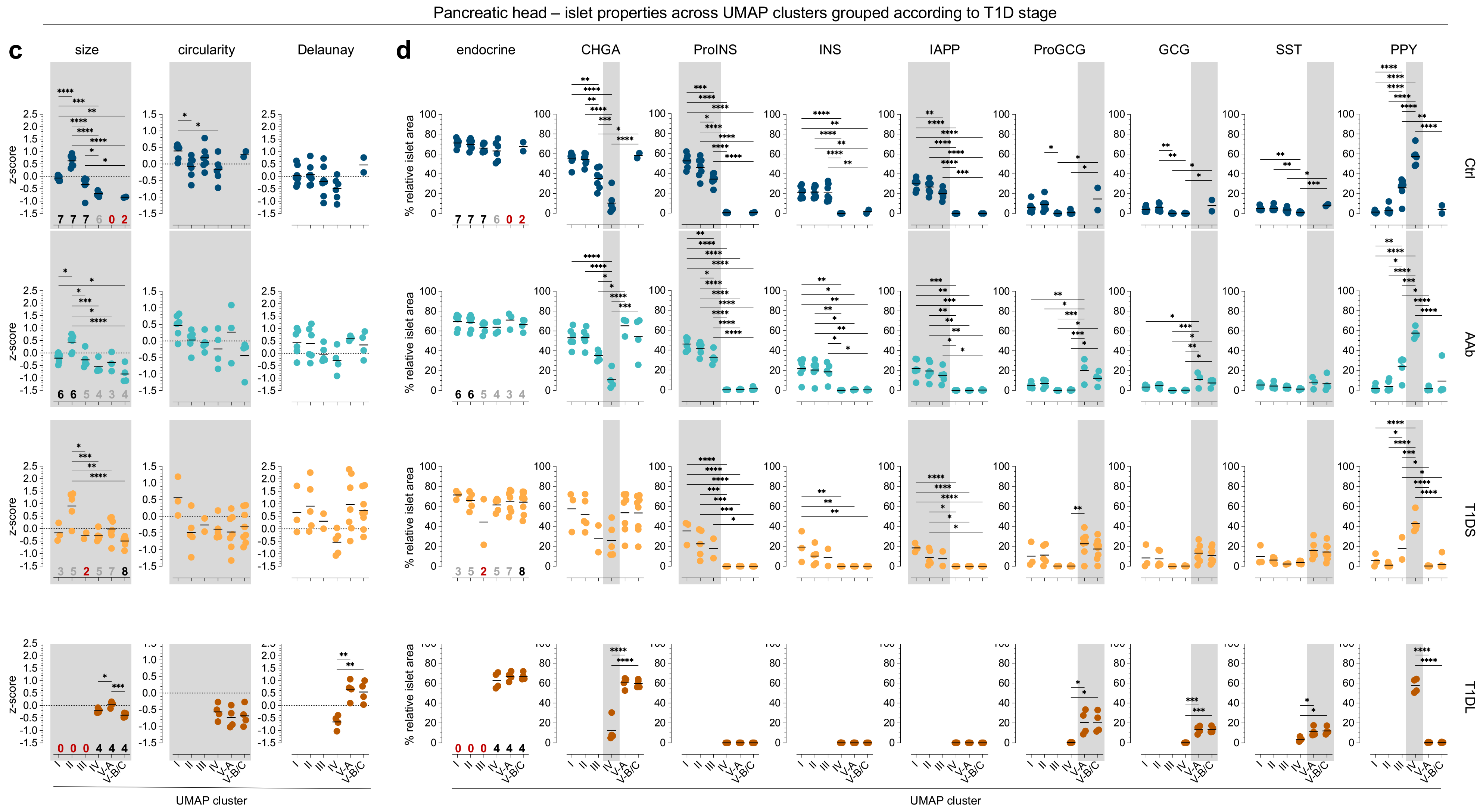

Figure S5c/d

Figure S5e/f

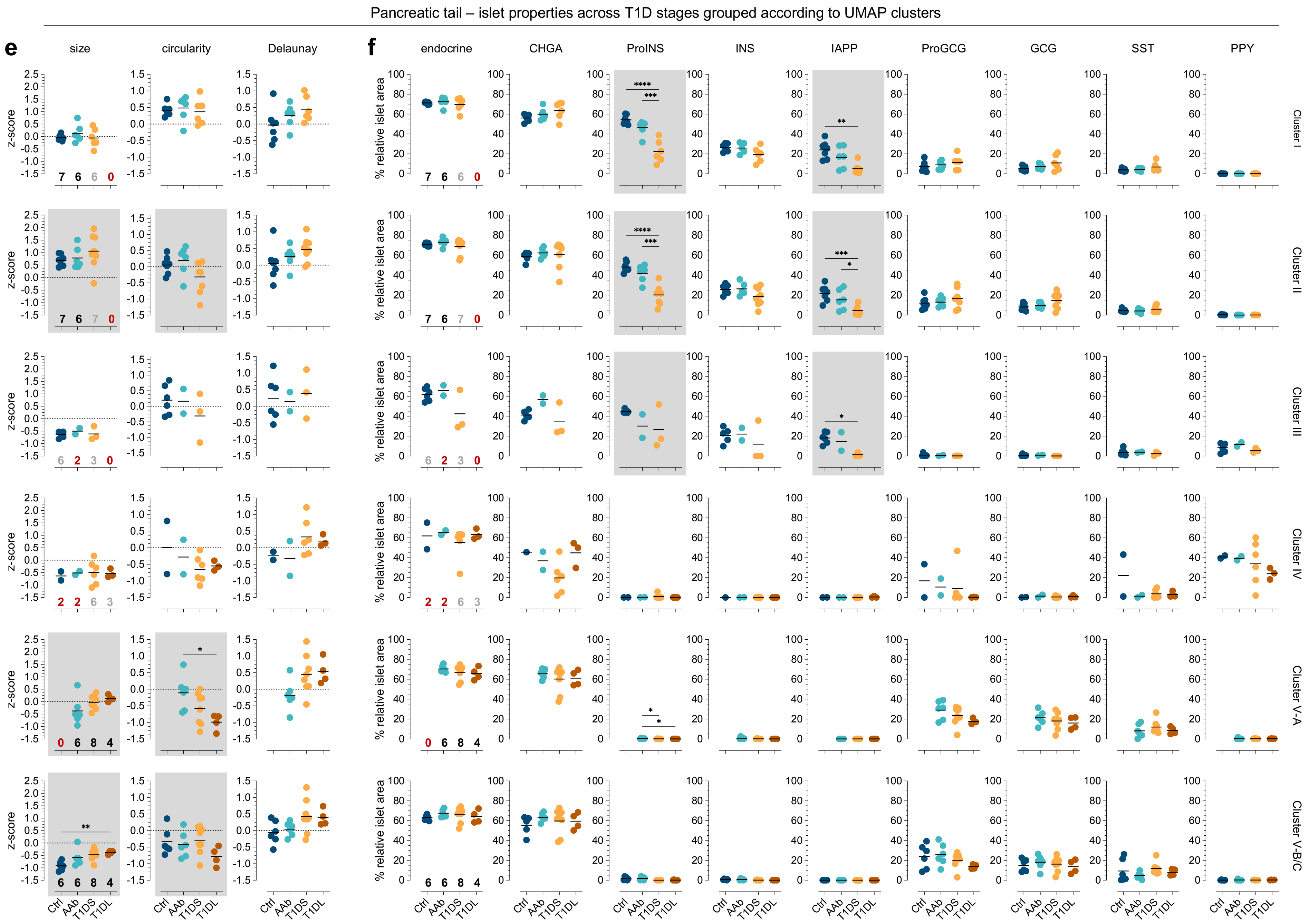

Figure S5e/f

Figure S5g/h

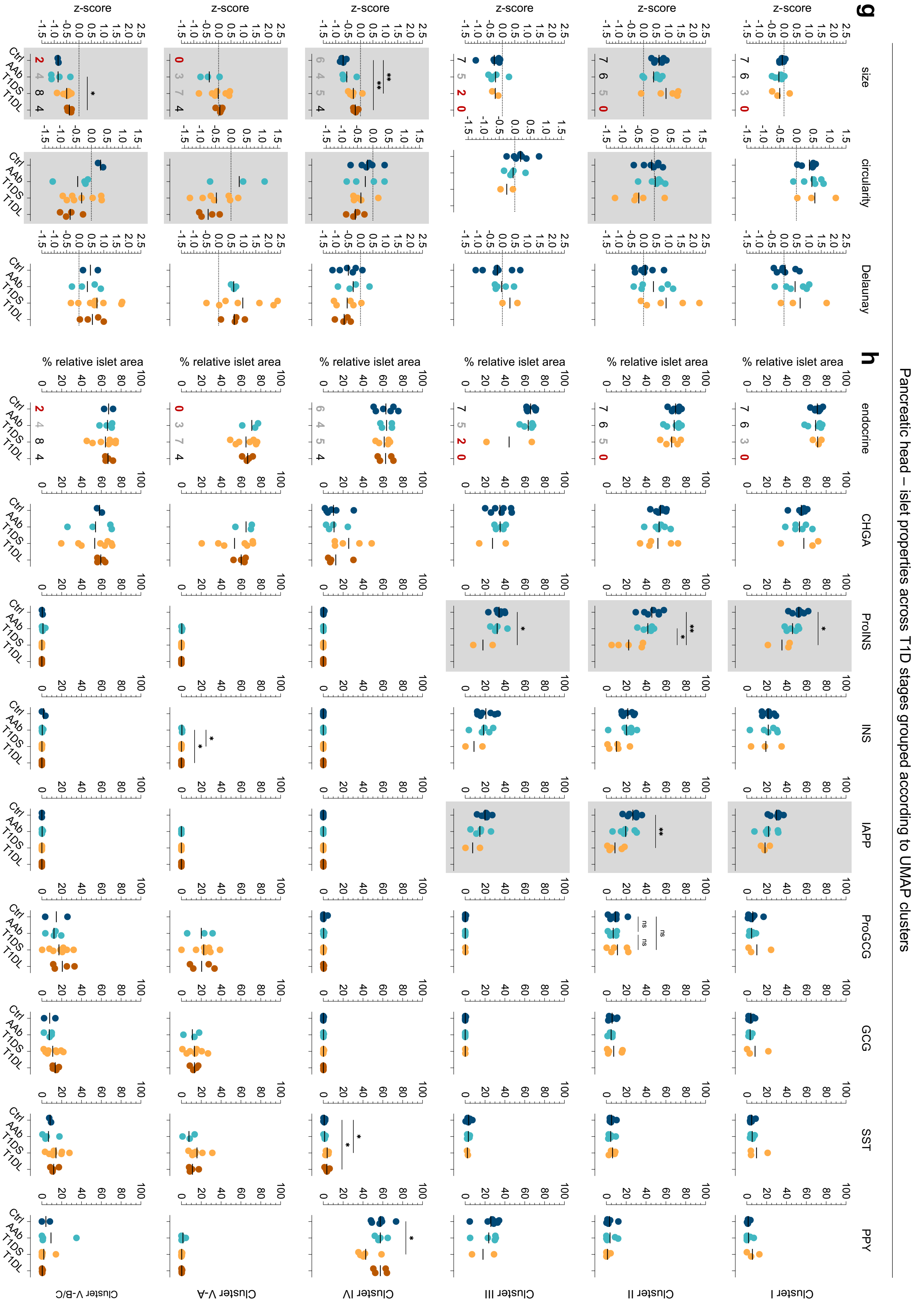

Figure S5g/h

Figure S5i/j

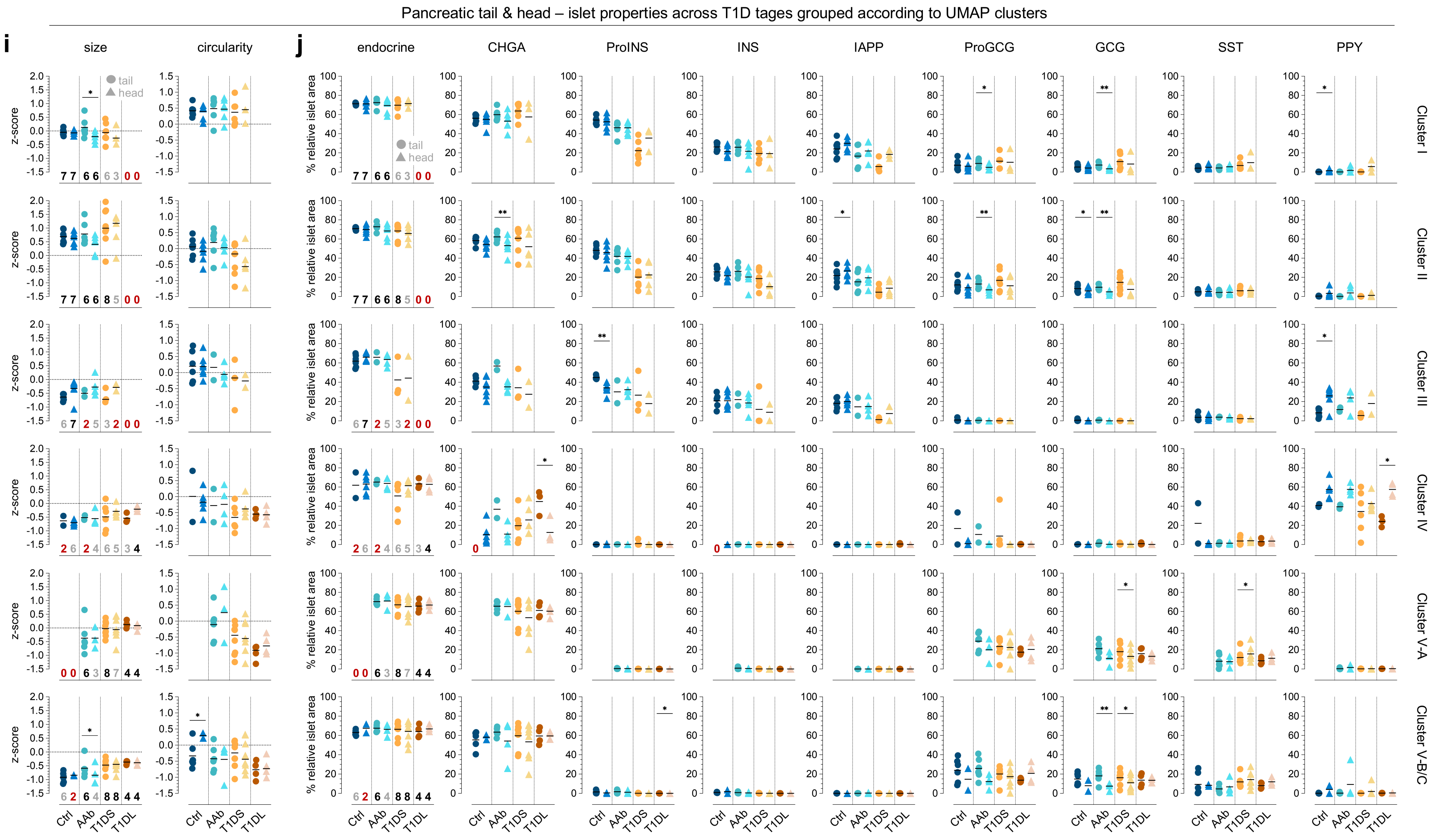

Figure S5i/j

Supplementary Figure S5. Consolidated data resource: islet properties across UMAP clusters and T1D stages. ***Fig.S5*** serves as a consolidated data resource, and islet features specifically called out in the main narrative are highlighted with gray backgrounds (all scatter plots display individual donor means [colored symbols] and group means [black horizontal bars]). The data is arranged into three sections allowing for statistical comparisons of islet properties between different UMAP clusters at each T1D disease stage (panels a-d); between the same UMAP clusters at different disease stages (e-h); and between PT and PH regions at each T1D stage and for each cluster (panels i/j). **a.-d.,** scatter plots compare respective analytical parameters (scatter and mean) between UMAP clusters I, II, III, IV, V-A and V-BC, and are grouped according to T1D stage (top to bottom rows). **a,** islet shape descriptors for PT (Delaunay z-scores refer to a distance property of individual islets in relation to neighboring islets as detailed in ***Fig.7d***); **b,** relative islet endocrine hormone expression in the PT ("endocrine" in the first column of panel b refers to the union of all hormone staining areas); **c,** islet shape descriptors for the PH; and **d,** relative islet endocrine hormone expression in the PH. **e.-h.,** the data featured in panels e-h are the same as in panels a-d but now all scatter plots compare islet properties between disease stages (Ctrl, AAb, T1DS, T1DL) and are grouped according to UMAP cluster affiliation (top to bottom rows). **e,** islet shape descriptors for PT; **f,** relative islet endocrine hormone expression in PT; **g,** islet shape descriptors for PH; and **h,** relative islet endocrine hormone expression in PH. **i./j.,** the data for UMAP cluster-resolved islet size, circularity and endocrine hormone expression featured in panels i/j is the same as in panels a-d but now scatter plots are arranged for a direct comparison of islet properties in PT *vs.* PH across T1D disease stages (Ctrl, AAb, T1DS, T1DL) and grouped according to UMAP cluster affiliation (top to bottom rows). Color-coded values on top of x-axes are the numbers of donors represented in each cluster and disease stage (black: all donors; gray: reduced number of donors; red: instances with 2 donors only warranting interpretative caution, or absent/excluded data), and are applicable to all plots in the same panel row. Statistical analyses were conducted with ordinary one-way ANOVA and Tukey's multiple comparisons test (panels a-h) or paired Student's t-test (two-tailed) for comparisons of PT *vs.* PH islet properties (panels i/j) adhering to the following convention: *p<0.05, **p<0.01, ***p<0.001, ****p<0.0001.

Figure S6

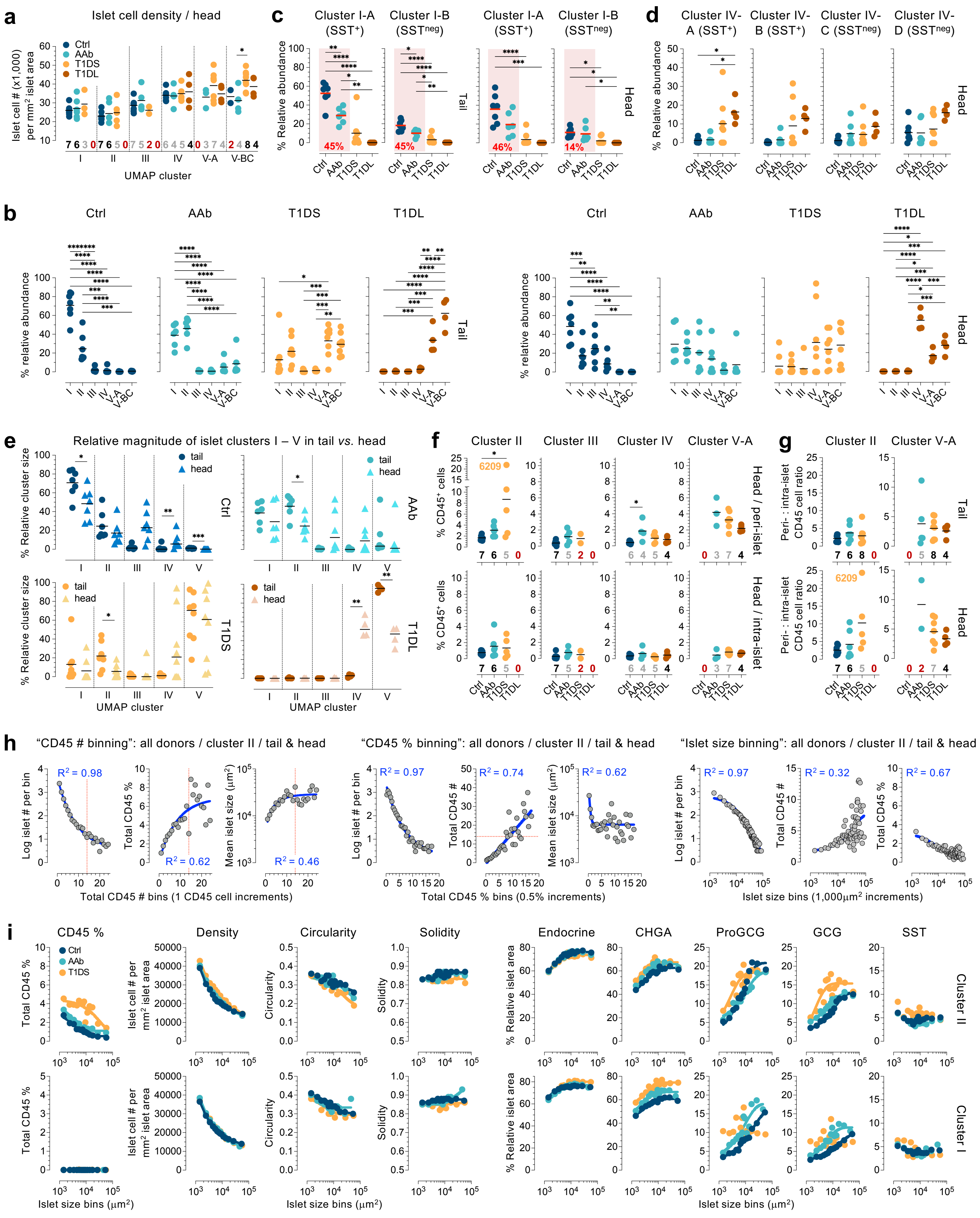

a
Islet cell density / head
Islet cell # (x1,000) per mm² islet area
Ctrl
AAb
T1DS
T1DL
UMAP cluster
c
Cluster I-A (SST+)
Cluster I-B (SSTneg)
% Relative abundance
Tail
Head
d
Cluster IV-A (SST+)
Cluster IV-B (SST+)
Cluster IV-C (SSTneg)
Cluster IV-D (SSTneg)
b
% relative abundance
e
Relative magnitude of islet clusters I – V in tail vs. head
% Relative cluster size
tail
head
f
Cluster II
Cluster III
Cluster IV
Cluster V-A
% CD45+ cells
Head / peri-islet
Head / intra-islet
g
Peri- : intra-islet CD45 cell ratio
h
"CD45 # binning": all donors / cluster II / tail & head
"CD45 % binning": all donors / cluster II / tail & head
"Islet size binning": all donors / cluster II / tail & head
Log islet # per bin
Total CD45 %
Mean islet size (µm²)
Total CD45 #
Total CD45 # bins (1 CD45 cell increments)
Total CD45 % bins (0.5% increments)
Islet size bins (1,000µm² increments)
i
CD45 %
Density
Circularity
Solidity
Endocrine
CHGA
ProGCG
GCG
SST
Islet cell # per mm² islet area
% Relative islet area
Cluster II
Cluster I
Islet size bins (µm²)

Supplementary Figure S6. UMAP islet cluster features, islet properties & CD45$^+$ cell infiltration. Unless noted otherwise, scatter plots display individual donor means (colored symbols) and group means (horizontal bars), **a.,** UMAP cluster-level islet cell densities in PH (all clusters I – III *vs.* IV – V-BC: p<0.0001). Note that in panels a, f and g not all donors have islets populating all clusters, and in addition we excluded values if <3 islets or <2 donors were represented in a cluster; the resultant numbers of donors represented in each cluster and disease stage is indicated by color-coded values above x-axes (black: all donors; gray: reduced number of donors; red: instances with 2 donors only warranting interpretative caution, or absent/excluded data). **b.,** relative UMAP cluster sizes in PT (left) and PH (right) within each donor group (note that ***Fig.4c*** displays the same data but is arranged according to disease stage within each UMAP cluster). **c.,** relative magnitude of UMAP islet subclusters I-A and I-B in PT and PH across T1D disease stages. To ascertain the collapse of cluster I from Ctrl to AAb stage in more detail, we considered its subclusters I-A/B/C where the main difference pertains to the presence (I-A) or absence (I-B) of delta cells (*cf.,* ***Fig.3c***) (the minor subcluster I-C is only found in the PH and contains islets with beta, gamma and delta but not alpha cells; *cf.,* ***Fig.3a/c***). Compared to Ctrl donors, AAb donors present with a 45% reduction in PT clusters I-A/B and PH cluster I-A as well as a lesser 15% decrease in PH cluster I-B. **d.,** relative magnitude of UMAP islet subclusters IV-A – D in the PH across T1D disease stages **e.,** relative magnitude of UMAP islet clusters I – V in PT *vs.* PH across T1D disease stages (*cf.,* cluster size comparisons in panel b and ***Fig.4c***). In aggregate, the consideration of donor-specific islet cluster size distributions permits the identification and characterization of several outliers: Ctrl 6454 presents with an "AAb-like" cluster II dominance that would appear consistent with their unusually high beta and alpha cell mass (*cf.,* ***Fig.S2k***); AAb 6310, despite being only single AAb$^+$ at time of death and with low genetic HLA risk, exhibits a pattern comparable to donors with multiple AAbs and thus may have been at greater T1D risk than clinically apparent; AAb 6450 has a profile resembling T1DS donors, moderate HLA risk and might have been poised to develop clinical disease in the near future; and T1DS 6405 resembles AAb donors and is distinct from other T1DS donors on account of severe obesity, excessive pancreas weight, elevated C-peptide and comparatively lower Hb1Ac despite high HLA risk (*cf.,* ***Fig.S2a-d***; for individual donor HLA risk scores, ***Supplementary Data 1***). **f.,** scatter plot summaries depicting peri- and intra-islet CD45$^+$ cell frequencies in PH clusters II – V-A. **g.,** peri-islet : intra-islet CD45$^+$ frequency ratios in clusters II and V-A of PT and PH. **h.,** correlations of total islet-associated CD45$^+$ cell numbers, CD45$^+$ cell frequencies and islet size in cluster II (PT/PH combined; gray circles indicate mean values of all donors). CD45$^+$ number binning was performed in one CD45$^+$ cell increments, CD45$^+$ cell percentage binning in 0.5% increments, and islet size binning employed 1,000 µm^2 increments; curve fits correspond to exponential associations and goodness of fit (R^2) is indicated (the vertical or horizontal red broken lines indicate the insulitis threshold of ≥15 CD45$^+$ cells). "CD45$^+$ # binning" documents a straightforward exponential association with corresponding CD45$^+$ cell frequencies that is particularly good for ~1-8 CD45$^+$ cells; similarly, "CD45$^+$ % binning" reveals a quasi-linear association that again is especially tight for islets with ~1-9 CD45$^+$ cells. However, these relations are not strictly reciprocal since they yield somewhat different CD45$^+$ cell frequencies corresponding to the insulitis threshold ("CD45$^+$ # binning": 5.7%; "CD45$^+$ % binning": 8.5%; also note that the curve fit for CD45$^+$ frequency bins *vs.* islet size ["CD45$^+$ % binning", right plot] demonstrates a brief exponential decline followed by a plateau starting at CD45$^+$ cell frequencies of ~1.5-2.0%). Notably, "islet size binning" yields a positive correlation of islet size with CD45$^+$ numbers but negative correlation with CD45$^+$ frequencies since small denominators inflate relative frequencies of CD45$^+$ cells even if their numbers are low. **i.,** combined PT and PH cluster II (top) and cluster I (bottom) islets stratified according to Ctrl, AAb or T1DS stage were "size-binned" for comparison of associated CD45$^+$ cell frequencies, shape descriptors and relative hormone expression areas (Ctrl cluster I: 5,706 islets, cluster II: 1,750 islets; AAb cluster I: 2,279 islets, cluster II: 2,426 islets; T1DS cluster I: 640 islets, cluster II: 745 islets; variable islet size bins [1,000-10,000µm^2: 9 bins, 10,000-15,000 µm^2: 2 bins, 15,000-20,000 µm^2: 1 bin, 20,000-40,000 µm^2: 1 bin, 40,000-100,000 µm^2: 1 bin]; each data point is the mean of all cluster II or cluster I islets within respective size bins; where applicable, curve fits represent exponential associations). Statistical analyses were conducted with n = 7 Ctrl, 6 AAb, 8 T1DS and 4 T1DL donors using ordinary one-way ANOVA and Tukey's multiple comparisons test (panels a-d, f and g) or paired Student's t-test (two-tailed; panel e) with *p<0.05, **p<0.01, ***p <0.001, ****p<0.0001.

Figure S7

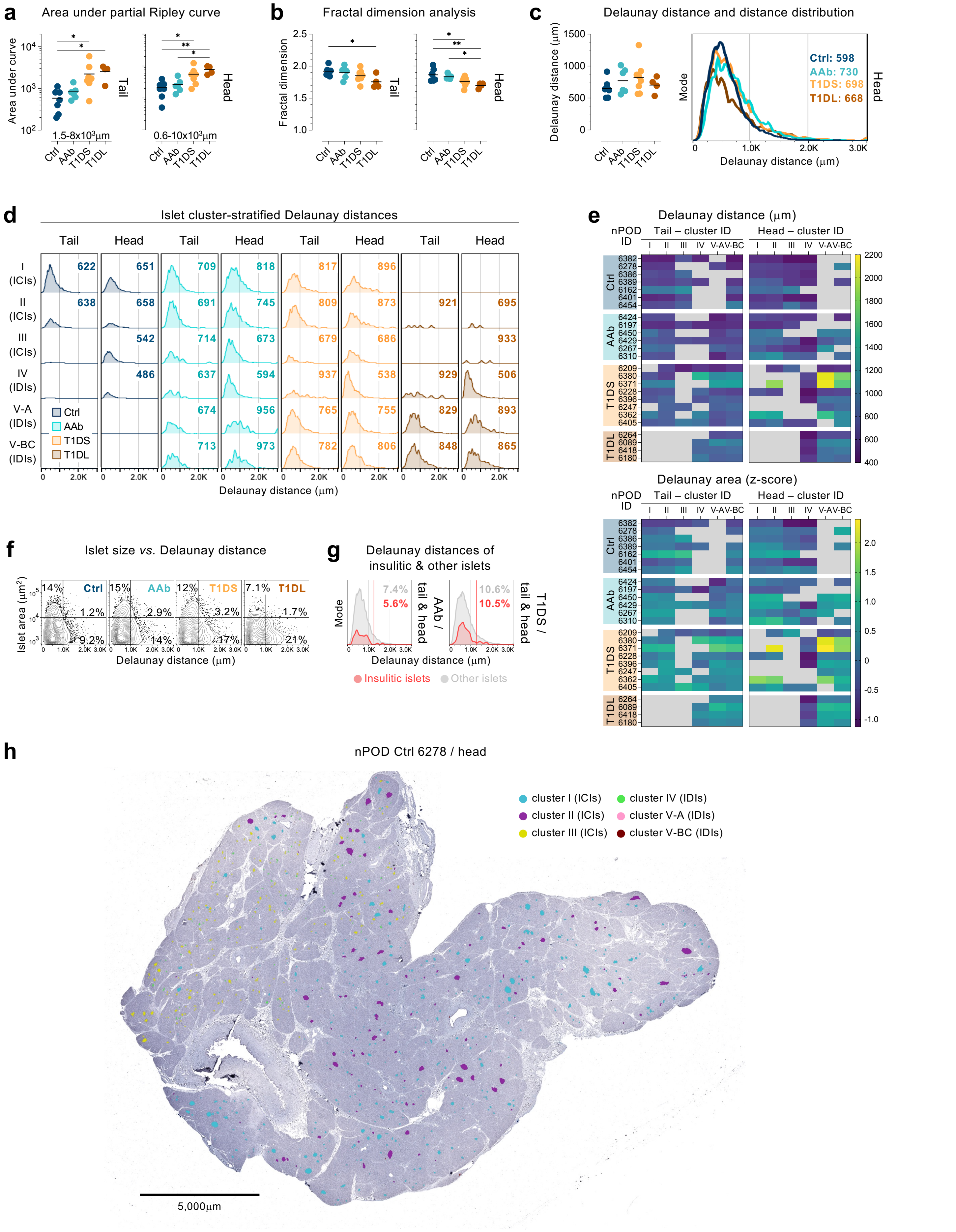
a
Area under partial Ripley curve
Area under curve
1.5-8x10³µm
0.6-10x10³µm
Ctrl
AAb
T1DS
T1DL
Tail
Head
b
Fractal dimension analysis
Fractal dimension
c
Delaunay distance and distance distribution
Delaunay distance (µm)
Mode
Ctrl: 598
AAb: 730
T1DS: 698
T1DL: 668
Delaunay distance (µm)
d
Islet cluster-stratified Delaunay distances
I (ICIs)
II (ICIs)
III (ICIs)
IV (IDIs)
V-A (IDIs)
V-BC (IDIs)
Delaunay distance (µm)
e
Delaunay distance (µm)
nPOD ID
Tail – cluster ID
Head – cluster ID
Delaunay area (z-score)
f
Islet size vs. Delaunay distance
Islet area (µm²)
Delaunay distance (µm)
g
Delaunay distances of insulitic & other islets
AAb / tail & head
T1DS / tail & head
Delaunay distance (µm)
Insulitic islets
Other islets
h
nPOD Ctrl 6278 / head
cluster I (ICIs)
cluster II (ICIs)
cluster III (ICIs)
cluster IV (IDIs)
cluster V-A (IDIs)
cluster V-BC (IDIs)
5,000µm

Supplementary Figure 7. Spatial distribution of UMAP-clustered islet subsets across pancreatic tissue sections and T1D disease stages (I). a., modified Ripley's K function analyses were conducted as detailed in ***Fig.7a-c*** and Methods. To assess differences in islet density enrichments across diseases groups, one-way ANOVA tests were performed for each distance bin and are significant for the range of 1.5-8x10^3 μm in PT, and 0.6-10x10^3 μm in PH (PH 6380 excluded due to very scarce islets, *cf.* ***Fig.S8a***); area under Ripley curve calculations displayed here only pertain to the above distance ranges. **b.,** islet distribution in each tissue section can also be described using fractal geometry (see Methods): boundary-adjusted islet counts found at each search radius were fit to a power function; each point here is the exponent calculated for a single slide; and a random distribution of islets would lead to a value of 2 while smaller values indicate section-wide density enrichments. **c.,** mean Delaunay distances between islets across, and distribution of mean Delaunay distances according to, T1D stages. **d.,** Delaunay distance distributions of islets grouped according to cluster affiliation and disease stage in PT and PH; values in each histogram indicate the corresponding mean Delaunay distances. Note that a marked rise of Delaunay distances for cluster I islets in AAb donors further increases with T1D onset; a similar pattern is observed for cluster II islets, and cluster III islets in the PH; and Delaunay distances for cluster V islets in the PT but not PH expand from AAb to T1DL stage. A notable exception pertains to cluster IV islets in the PH which exhibit an overall greater proximity that appears to grow even closer with T1D progression; this likely results from a combination of unique regionalization, an imperviousness to T1D pathological processes due to constitutive lack of beta cells, and an incorporation of former cluster III islets rendered beta cell-deficient (*cf.,* ***Fig.4c/f***). **e.,** heatmaps stratifying Delaunay distances (top) and Delaunay area z-scores (bottom) across T1D stage, individual donors, islet cluster affiliation, and PT/PH regions (missing/excluded values in gray); Delaunay area z-scores are also featured in the summary ***Fig.S5a/c/e/g***. **f.,** contour plots are gated on combined islets within all donor groups and display islet size against mean Delaunay distance; quadrant markers distinguish larger islets (>10,000 μm^2) and greater Delaunay distances (>1,000 μm), and values are percentages of islets in each quadrant. Note that both larger and smaller islets are subject to the disease-associated increase of Delaunay distances. **g.,** Delaunay distances for insulitic and other islets (histograms gated on combined PT/PH islets from AAb *vs.* T1DS donors; values are the respective fractions of islets at >1,250 μm Delaunay distance). Both non-insulitic and insulitic islets display essentially similar neighborhood relations with small subsets (~6% in AAb, ~11% in T1DS) that are more "isolated" at a distance of >1,250 μm. **h.,** *in situ* projection of islets color-coded according to cluster affiliation onto a representative PH section. Scatter plots in panels a-c display individual donor means (colored circles) and group means (black horizontal bars); statistical analyses were conducted with n = 7 Ctrl, 6 AAb, 8 T1DS and 4 T1DL donors using ordinary one-way ANOVA and Tukey's multiple comparisons test with *p<0.05 and **p<0.01.

Figure S8

a

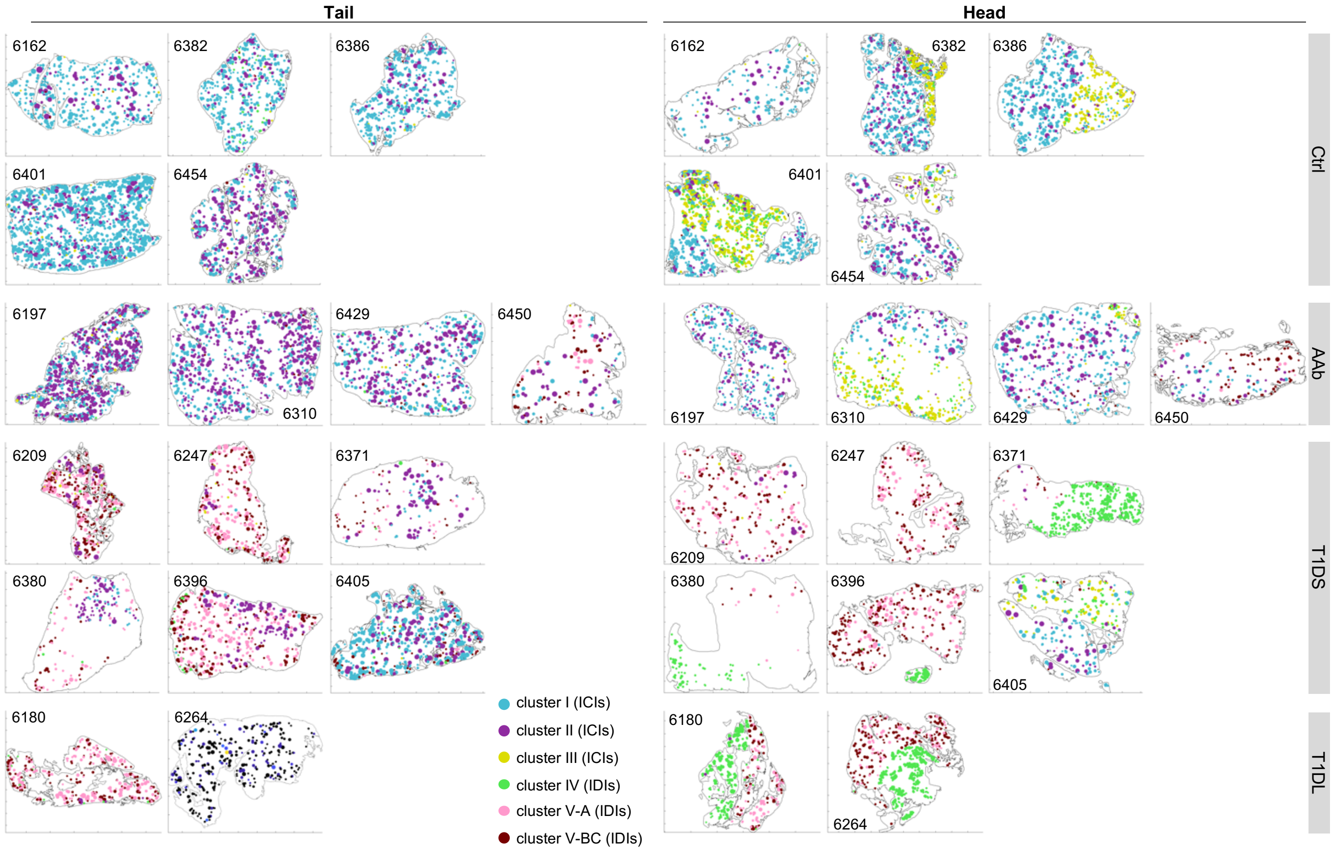

b

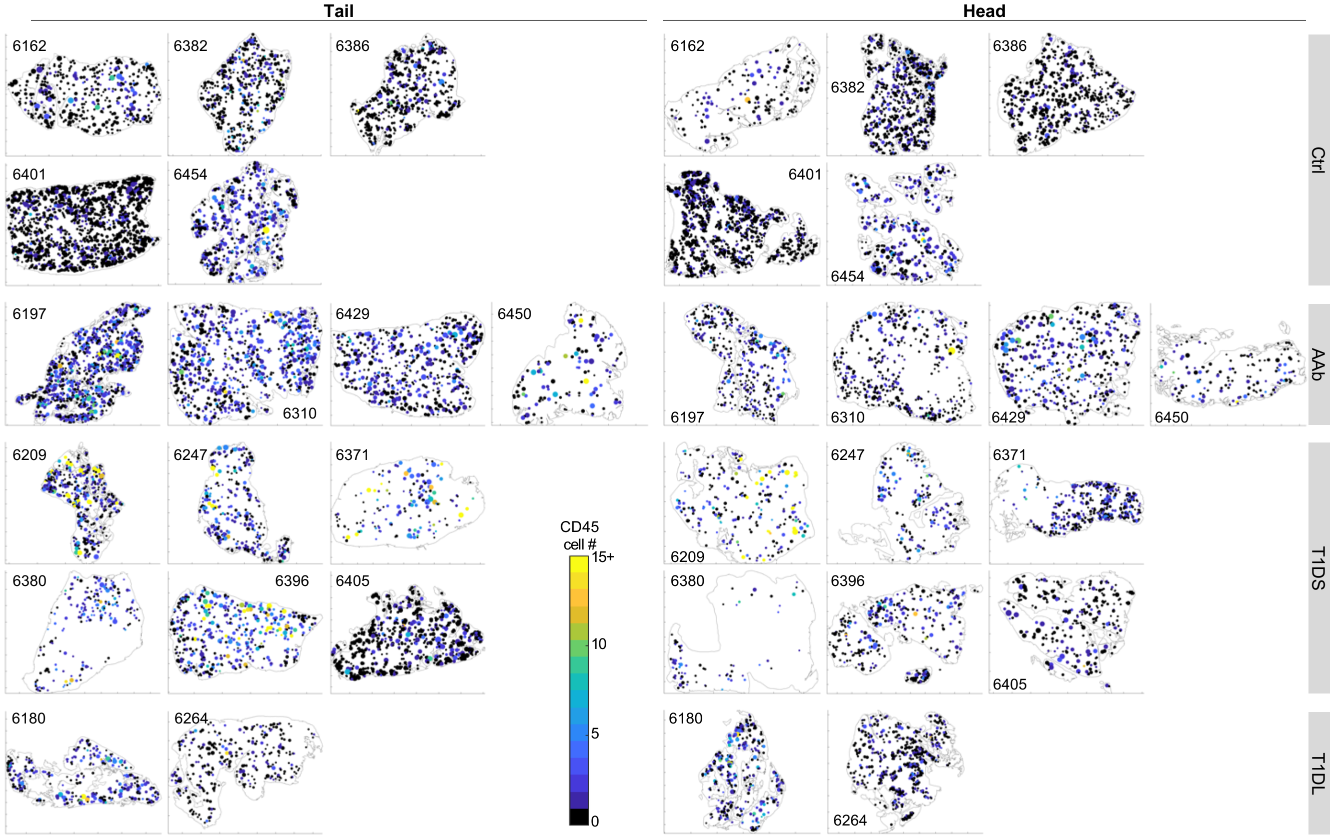

c Approximation of “islet clustering” with low dimensionality immunostaining

	INS	GCG	peri- & intra islet CD45	PPY
cluster I	+	+ or -	0	-
cluster II	+	+ or -	≥ 1	-
cluster III	+	-	≥ 0	+
cluster IV	-	-	≥ 0	+
cluster V-A	-	+	≥ 1	-
cluster V-BC	-	+	0	-

Supplementary Figure 8. Spatial distribution of UMAP-clustered islet subsets across pancreatic tissue sections and T1D disease stages (II). a. & b., pancreatic tissue section outlines (regions, T1D stage and nPOD donor IDs are indicated) populated with color-coded islets; for facilitated visual presentation, all islets are rendered circular and enlarged with relative size differences preserved. **a,** islets color-coded according to UMAP cluster affiliation. **b,** islets color-coded according to the numbers (0-15+) of associated $CD45^+$ cells. Distinctive histopathological properties of three "outlier" cases are readily visualized in this data display: AAb 6450 with a histopathological presentation that appears "T1DS-like"; AAb 6310 who despite single AAb positivity features islet subset distributions similar to AAb donors with ≥2 AAbs (*cf.,* ***Fig.4b***); and T1DS 6405, the donor with severe obesity and better glucose handling (*cf.,* ***Fig.S2b/c***) whose pancreas histology resembles AAb or even Ctrl donors. **c.,** approximation of islet cluster stratification with only INS, GCG and CD45 stains; in the absence of additional PPY stains, cluster III islets in the PH cannot be distinguished from GCG^{neg} cluster I or II islets, and cluster IV islets remain "invisible".

Table S1. Antibodies and MICSSS staining conditions.

Target	Clone	Species & isotype	Vendor	Staining condition	2nd Ab staining condition	HRP-SAV
Proinsulin (ProINS)	GS-9A8	Mouse (Ms) IgG1	Novo Nordisk; also available from DHSB #GS-9A8	1:200<br>1h, RT	1:800<br>30min, RT	1:300<br>30min, RT
Islet amylin (IAPP)	polyclonal	Rabbit (Rb) IgG	Sigma<br>#HPA053194	1:1000<br>1h, RT	1:1000<br>30min, RT	1:300<br>30min, RT
Chromogranin A (CHGA)	LK2H10+PHE5	Ms IgG1	Novus Biologicals<br>#NBP2-34239	1:150<br>1h, RT	1:850<br>30min, RT	1:300<br>30min, RT
Glucagon (GCG)	IMD-7	Ms IgG1	Abcam<br>#ab82270; discontinued, available from LS Bio #LS C171152	1:500,<br>1h, RT	1:850<br>30min, RT	1:300<br>30min, RT
Insulin (INS)	polyclonal	Guinea pig (Gp) IgG	DAKO<br>#A0564; discontinued, now only available as ready-to-use format #IR00261-2	1:850<br>1h, RT	1:1000<br>30min, RT	1:300<br>30min, RT
Somatostatin (SST)	polyclonal	Rb IgG	DAKO<br>#A0566; discontinued, alternative available from Genetex #GTX60646 (Ms IgG1; clone 7G5)	1:350<br>1h, RT	1:750<br>30min, RT	1:300<br>30min, RT
Pancreatic polypeptide (PPY)	polyclonal	Goat IgG	Novus<br>#NB100-1793	1:100<br>1.5h, RT	1:250<br>30min, RT	1:300<br>30min, RT
Proglucagon (ProGCG)	D16G10	Rb IgG	Cell Signaling<br>#8233	1:75<br>1.5h RT	1:300<br>30min, RT	1:300<br>30min, RT
CD45	2B11 + PD7/26	Ms IgG1	DAKO<br>#M0701	1:100<br>2h, RT	1:250<br>30min RT	1:300<br>30min, RT

Ab, antibody; RT, room temperature; HRP-SAV, horseradish peroxidase-conjugated streptavidin

SUPPLEMENTARY REFERENCES

1. Achenbach, P., *et al.* Characteristics of rapid vs slow progression to type 1 diabetes in multiple islet autoantibody-positive children. *Diabetologia* **56**, 1615-1622 (2013).

2. Campbell-Thompson, M.L., *et al.* Collection protocol for human pancreas. *Journal of Visualized Experiments: JoVE* **63**, e4039 (2012).

3. Rodriguez-Calvo, T., *et al.* Increase in Pancreatic Proinsulin and Preservation of Beta Cell Mass in Autoantibody Positive Donors prior to Type 1 Diabetes Onset. *Diabetes* **66**, 1334-1345 (2017).

4. Wasserfall, C., *et al.* Persistence of Pancreatic Insulin mRNA Expression and Proinsulin Protein in Type 1 Diabetes Pancreata. *Cell Metab* **26**, 568-575 e563 (2017).

5. Sims, E.K., *et al.* Abnormalities in proinsulin processing in islets from individuals with longstanding T1D. *Translational Research: The Journal of Laboratory and Clinical Medicine* **213**, 90-99 (2019).

6. Rodriguez-Calvo, T., *et al.* Altered beta-Cell Prohormone Processing and Secretion in Type 1 Diabetes. *Diabetes* **70**, 1038-1050 (2021).

7. Leete, P., *et al.* Studies of insulin and proinsulin in pancreas and serum support the existence of aetiopathological endotypes of type 1 diabetes associated with age at diagnosis. *Diabetologia* **63**, 1258-1267 (2020).

8. Lehrstrand, J., *et al.* Illuminating the complete beta-cell mass of the human pancreas - signifying a new view on the islets of Langerhans. *Nature Communications* **15**, 3318 (2024).
