# Supplementary material for: Integrated histopathology of the human pancreas throughout stages of type 1 diabetes progression
Source: Nat Commun. 2026 Feb 11;17:4293. doi: 10.1038/s41467-026-68610-1 (PMC13168456; doi:10.1038/s41467-026-68610-1)
Supplement: Supplementary file 2 — Description of Additional Supplementary Files [file 41467_2026_68610_MOESM2_ESM.docx]

**Integrated histopathology of the human pancreas**

**throughout stages of type 1 diabetes progression**

*Verena van der Heide, Sara McArdle, Michael S. Nelson, Karen Cerosaletti, Sacha Gnjatic,*

*Zbigniew Mikulski, Amanda L. Posgai, Irina Kusmartseva, Mark A. Atkinson & Dirk Homann*

**DESCRIPTION OF ADDITIONAL SUPPLEMENTARY FILES**

**Supplementary Data 1. Pancreas specimen information and donor metadata** (provided as a separate .xlsx file)**.** All pancreatic tissue sections were obtained from the Network for Pancreatic Organ Donors with Diabetes (nPOD). ***Supplementary Data 1*** summarizes pancreas donor information arranged into four groups (Ctrl, AAb, T1DS, T1DL; within each group, donors are listed in order of increasing age) and comprises nPOD donor ID; formalin-fixed, paraffin-embedded (FFPE) tissue block details; donor demographics (gender, ethnicity, age, age at T1D onset, T1D duration); clinical parameters (body mass index, C-peptide, HbA1c, number and type of AAbs, cause of death); pathology assessments (pancreas weight, presence/absence of insulitis); and genetics (available HLA haplotypes were used in conjunction with ethnicity to calculate genetic T1D risk as detailed in Methods).

***Footnotes*:** Letters at end of case ID & block # (A, B, C) for 18 out of 50 sections indicate partial rather than complete pancreatic cross-sections (for standard nPOD pancreas processing protocols and generation/cutting of tissue blocks, see PMID: 22665046). T1D staging for AAb donors is based solely on the number of autoantibodies and, in the absence of metabolic tests, cannot distinguish between stage 1 and 2 T1D. AA: African American; AMR: Admixed American (Hispanic/Latino); CEU: Caucasians of European descent. All numerical values in columns AD, AE, AG and AI represent odds ratios. n/a: not applicable; na: not available. * insulitis assessment: the column features results from manual assessments conducted by nPOD pathologists. Our semi-automated image analyses largely reproduced these assessments yet we did not confirm insulitis cases described earlier as “low-grade” (AAb 6310; four islets with 51, 16, 14 and 14 CD45 cells, respectively) or “possibly” (T1DS 6380), nor for T1DL 6264 where insulitis was recorded in the pancreas body, a region not interrogated here. While we identified additional insulitis cases in PH sections from AAb 6424 and T1DL 6180 (*cf.,* ***Fig.5a***), the only gross diagnostic discordance pertains to T1DS 6405, a severely obese donor for whom “insulitis present in all regions” is noted in the nPOD case report (visual inspection confirmed abundant CD45^+^ cells in exocrine tissue of this donor yet few were associated with islets).

**Supplementary Data 2. Properties of individual donor tissue sections and islets captured therein** (provided as a separate .xlsx file)**. *Supplementary Data 2*** features properties of donor tissue sections, and of all ~25,000 individual islets captured in the present study and stratified according to pancreas region (PT, PH), donor group (Ctrl, AAb, T1DS, T1DL; including associated de-identified demographic and clinical metadata), and UMAP sub/cluster affiliation (I - V-BC).

***Footnotes*:** Parenchymal tissue area (mm^2^): *cf.,* ***Fig.S2e***; parenchymal / total tissue area (%): *cf.,* ***Fig.S2e***; islet # / section: *cf.,* ***Fig.S2g***; islet density (islet # / mm^2^ parenchymal area): *cf.,* ***Figs.1i & S2h****.*
