# Supplementary material for: Integrated histopathology of the human pancreas throughout stages of type 1 diabetes progression
Source: Nat Commun. 2026 Feb 11;17:4293. doi: 10.1038/s41467-026-68610-1 (PMC13168456; doi:10.1038/s41467-026-68610-1)
Supplement: Supplementary file 5 — Reporting Summary [file 41467_2026_68610_MOESM5_ESM.pdf]

nature portfolio

Corresponding author(s): Dirk Homann

Last updated by author(s): November 19, 2025

Reporting Summary

Nature Portfolio wishes to improve the reproducibility of the work that we publish. This form provides structure for consistency and transparency in reporting. For further information on Nature Portfolio policies, see our Editorial Policies and the Editorial Policy Checklist.

Statistics

For all statistical analyses, confirm that the following items are present in the figure legend, table legend, main text, or Methods section.

n/a	Confirmed	
☐	☒	The exact sample size (*n*) for each experimental group/condition, given as a discrete number and unit of measurement
☐	☒	A statement on whether measurements were taken from distinct samples or whether the same sample was measured repeatedly
☐	☒	The statistical test(s) used AND whether they are one- or two-sided<br>*Only common tests should be described solely by name; describe more complex techniques in the Methods section.*
☐	☒	A description of all covariates tested
☐	☒	A description of any assumptions or corrections, such as tests of normality and adjustment for multiple comparisons
☐	☒	A full description of the statistical parameters including central tendency (e.g. means) or other basic estimates (e.g. regression coefficient) AND variation (e.g. standard deviation) or associated estimates of uncertainty (e.g. confidence intervals)
☐	☒	For null hypothesis testing, the test statistic (e.g. *F*, *t*, *r*) with confidence intervals, effect sizes, degrees of freedom and *P* value noted<br>*Give P values as exact values whenever suitable.*
☒	☐	For Bayesian analysis, information on the choice of priors and Markov chain Monte Carlo settings
☒	☐	For hierarchical and complex designs, identification of the appropriate level for tests and full reporting of outcomes
☐	☒	Estimates of effect sizes (e.g. Cohen's *d*, Pearson's *r*), indicating how they were calculated

Our web collection on statistics for biologists contains articles on many of the points above.

Software and code

Policy information about availability of computer code

Data collection	-Hamamatsu NDP.view2 (https://www.hamamatsu.com/us/en/product/life-science-and-medical-systems/digital-slide-scanner/U12388-01.html)
Data analysis	-QuPath (0.2.3 and 0.5.1)<br>-Matlab (2024a)<br>-CytoMAP (https://github.com/DrStoltzfus/CytoMAP)<br>-FlowJo 10.10.0<br>-GraphPad Prism (9 and 10)<br>-Custom scripts available at https://github.com/saramcardle/MICSSSPancreas

For manuscripts utilizing custom algorithms or software that are central to the research but not yet described in published literature, software must be made available to editors and reviewers. We strongly encourage code deposition in a community repository (e.g. GitHub). See the Nature Portfolio guidelines for submitting code & software for further information.

Data

Policy information about availability of data

All manuscripts must include a data availability statement. This statement should provide the following information, where applicable:

- Accession codes, unique identifiers, or web links for publicly available datasets
- A description of any restrictions on data availability
- For clinical datasets or third party data, please ensure that the statement adheres to our policy

The supplemental information provided for this study includes detailed pancreas specimen information and donor metadata (Supplementary Data 1); properties of individual donor tissue sections and all ~25,000 islets captured therein and stratified according to pancreas region, donor group and UMAP sub/cluster affiliation (Supplementary Data 2); details for antibodies and MICSSS staining conditions (Table S1), and source data for all main and supplemental figures (source data file). Raw whole-slide brightfield images of pancreatic tissue sections captured at 40x will be provided by the lead contact upon request.

Research involving human participants, their data, or biological material

Policy information about studies with human participants or human data. See also policy information about sex, gender (identity/presentation), and sexual orientation and race, ethnicity and racism.

Reporting on sex and gender	The present work is classified as "Not Human Subjects Research"; gender of all tissue donors as well as additional demographic and clinical variables are detailed in Supplementary Data 1 of the manuscript.
Reporting on race, ethnicity, or other socially relevant groupings	The present work constitutes "Not Human Subjects Research"; race/ethnicity of all tissue donors as well as additional demographic and clinical variables are detailed in Supplementary Data 1 of the manuscript.
Population characteristics	Not applicable.
Recruitment	Not applicable
Ethics oversight	This work uses pancreatic FFPE tissue sections procured from the Network for Pancreatic Organ Donors with Diabetes (nPOD; https://npod.org/for-investigators/request-npod-samples/). Informed consent for tissue usage as well as associated de-identified donor metadata (detailed in Supplementary Data 1) were obtained by the nPOD consortium, in accordance with Institutional Review Board procedures at the Icahn School of Medicine at Mount Sinai.

Note that full information on the approval of the study protocol must also be provided in the manuscript.

Field-specific reporting

Please select the one below that is the best fit for your research. If you are not sure, read the appropriate sections before making your selection.

[x] Life sciences [ ] Behavioural & social sciences [ ] Ecological, evolutionary & environmental sciences

For a reference copy of the document with all sections, see nature.com/documents/nr-reporting-summary-flat.pdf

Life sciences study design

All studies must disclose on these points even when the disclosure is negative.

Sample size	Sample size for the present study was contingent on availability of suitable pancreatic tissue sections covering the natural history of T1D development and progression (all tissue sections provided by the nPOD consortium). In specific, the study included pancreatic tail and head sections from 7 non-diabetic donors (Ctrl), 6 autoantibody-positive donors (AAb), 8 donors with short T1D duration (<2 years; T1DS), and 4 donors with long T1D duration (8-11 years; T1DL).
Data exclusions	For the purpose of the present study, pancreatic islets are defined as endocrine objects ≥1,000 µm (~36 µm diameter); accordingly, we excluded smaller endocrine structures and single cells from all of our analyses. Information about HLA haplotypes and HbA1c values was available for most but not all donors (Supplementary Data 1 & Fig.S2a/c). Subcluster IV-E (Fig.3a) is sample-biased and was not further considered. In some analyses of islet size frequency distributions and UMAP cluster-stratified islets, not all donors have islets present in all islet size bins or clusters; for islet property analyses, we additionally excluded values if <3 islets or <2 donors were represented in a size bin (Figs.2m-q & S4a-h) or UMAP cluster. Chromogranin A and insulin staining of Ctrl 6162 pancreatic tail tissue sections and insulin staining of AAb 6450 pancreatic tail sections was notably weak; in the absence of chromogranin A and insulin staining irregularities in the corresponding pancreatic head sections as well as normal proinsulin and islet amyloid polypeptide staining in both pancreatic tail and head sections, we attribute this observation to a technical staining issue and therefore excluded the respective pancreatic tail chromogranin A and insulin data from Figs.1k, 2p/q, 3d, S1d, S2i-k, S3a/b/f & S5b/f/j. For calculation of endocrine cell type mass, the two 5-year-old donors Ctrl 6382 and T1DS 6209 were excluded in Figs.1j & S2k, and the pancreatic head data from T1DS 6380 was not included in modified Ripley's K analyses due to very scarce islets (Figs.7c & S8a).
Replication	All experimental tissue sections were subjected to the iterative MICSSS process, and no replication of experimental procedures was possible due to limited availability of pancreatic tissue sections.
Randomization	In the absence of investigator blinding (see below), inter-experimental variability was reduced by executing each round of MICSSS staining in

three batches stained on three consecutive days (PT: pancreatic tail; PH: pancreatic head; numbers are nPOD case IDs):

Batch 1 - PT Ctrl: 6162, 6278, 6382; PT AAb: 6310, 6424, 6429, 6450; PT T1DS: 6209, 6228, 6247, 6362, 6380, 6371; PT T1DL: 6089, 6180, 6264.

Batch 2 - PT Ctrl: 6389, 6401, 6454, 6386; PT AAb: 6267, 6197; PT T1DS: 6396, 6405; PT T1DL: 6418; PH Ctrl: 6162; PH AAb: 6197, 6267; PH T1DS: 6209, 6228; 6247; PH T1DL: 6089, 6180.

Batch 3 - PH Ctrl: 6278, 6382, 6386, 6389, 6401, 6454; PH AAb: 6310, 6424, 6429, 6450; PH T1DS: 6362, 6371, 6380, 6396, 6405; PH T1DL: 6264, 6418.

Blinding

Investigator blinding to group allocation during data collection and analysis was not feasible for the present study.

Data analysis: the data output of 450 high-resolution whole-slide images was used to both develop, adjust and apply a semi-automated image analysis pipeline for the quantification of the features of ~25,000 individual islets across all donor cohorts and pancreas regions. Based on analytical requirements (e.g., overlay of multiple immuno-stained images from same donor/pancreas region; need for manual/visual assessment of provisional pipeline outputs; overall small sample size typical for T1D histopathology studies), investigator blinding was not possible.

Reporting for specific materials, systems and methods

We require information from authors about some types of materials, experimental systems and methods used in many studies. Here, indicate whether each material, system or method listed is relevant to your study. If you are not sure if a list item applies to your research, read the appropriate section before selecting a response.

Materials & experimental systems

n/a	Involved in the study
☐	☒ Antibodies
☒	☐ Eukaryotic cell lines
☒	☐ Palaeontology and archaeology
☒	☐ Animals and other organisms
☒	☐ Clinical data
☒	☐ Dual use research of concern
☒	☐ Plants

Methods

n/a	Involved in the study
☒	☐ ChIP-seq
☒	☐ Flow cytometry
☒	☐ MRI-based neuroimaging

Antibodies

Antibodies used

Target antigens were revealed by staining with primary antibodies; secondary antibodies (donkey-raised biotinylated F(ab')2 with minimal cross-reactivity [Jackson ImmunoResearch]); incubation with horseradish peroxidase (HRP)-conjugated streptavidin (HRP-SAV; DAKO, Agilent); and ImmPACT AMEC Red substrate (Vector Laboratories). Precise details about antibody staining protocols are found in the Methods section of the manuscript as well as in Supplementary Table S1.

Antibodies and staining conditions (listed in order of iterative MICSSS staining sequence):

-Proinsulin (ProINS); clone GS-9A8; Mouse (Ms) IgG1; Novo Nordisk (also available from DHSB); primary antibody staining: 1:200, 1h, room temperature (RT); secondary antibody staining: 1:800, 30min, RT; HRP-SAV: 1:300, 30min, RT.

-Islet amylin (IAPP); polyclonal Rabbit (Rb) IgG; Sigma #HPA053194; primary antibody staining: 1:1000, 1h, RT; secondary antibody staining: 1:1000, 30min, RT; HRP-SAV: 1:300, 30min, RT.

-Chromogranin A (CHGA); double clone LK2H10+PHE5; Ms IgG1; Novus Biologicals #NBP2-34239; primary antibody staining: 1:150, 1h, RT; secondary antibody staining: 1:850, 30min, RT; HRP-SAV: 1:300, 30min, RT.

-Glucagon (GCG); clone IMD-7; Ms IgG1; Abcam #ab82270 (discontinued, available from LS Bio #LS C171152); primary antibody staining: 1:500, 1h, RT; secondary antibody staining: 1:850, 30min, RT; HRP-SAV: 1:300, 30min, RT.

-Insulin (INS); polyclonal Guinea pig (Gp) IgG; DAKO #A0564 (discontinued, now only available as ready-to-use format #IR00261-2); primary antibody staining: 1:850, 1h, RT; secondary antibody staining: 1:1000, 30min, RT; HRP-SAV: 1:300, 30min, RT.

-Somatostatin (SST); polyclonal Rb IgG; DAKO #A0566 (discontinued, alternative available from Genetex #GTX60646; Ms IgG1; clone 7G5); primary antibody staining: 1:350, 1.5h, RT; secondary antibody staining: 1:750, 30min, RT; HRP-SAV: 1:300, 30min, RT.

-Pancreatic polypeptide (PPY); polyclonal Goat IgG; Novus #NB100-1793; primary antibody staining: 1:100, 1.5h, RT; secondary antibody staining: 1:250, 30min, RT; HRP-SAV: 1:300, 30min, RT.

-Proglucagon (ProGCG); clone D16G10; Rb IgG; Cell Signaling #8233; primary antibody staining: 1:75, 1.5h, RT; secondary antibody staining: 1:300, 30min, RT; HRP-SAV: 1:300, 30min, RT.

-CD45; double clone 2B11+PD7/26; Ms IgG1; DAKO #M0701; primary antibody staining: 1:100, 2h, RT; secondary antibody staining: 1:300, 30min, RT; HRP-SAV: 1:300, 30min, RT.

Validation

The ProINS antibody (clone GS-9A8) was deposited by Dr. O. Madsen in the DSHB (https://dshb.biology.uiowa.edu/GS-9A8; RRID:AB_532383) and provided to us in purified form by Dr. Madsen/NovoNordisk.

The IAPP antibody (Sigma #HPA053194; RRID:AB_2682076) is part of the stringently validated/characterized "Prestige Antibodies® Powered by Atlas Antibodies" portfolio.

The CHGA-specific double clone (Novus #NBP2-34239; RRID:AB_3285666) has been validated for use in FFPE tissue section staining (e.g., PMID: 27035983, PMID: 41053097).

The GCG antibody (abcam #ab82270; RRID:AB_1658481) was generated by immunization with polymerized porcine glucagon (detailed immunogen information proprietary) and has been validated for use in FFPE tissue section staining (e.g., PMID: 27506584).

The antibodies for INS (DAKO #A0564; RRID:AB_10013624), SST (DAKO #A0566; RRID:AB_2688022) and CD45 (DAKO #M0701; RRID:AB_2750582) have been previously validated by nPOD investigators (https://npod.org/wp-content/uploads/2021/05/OPPC-SOP-72.1-Immunopathology-1.pdf).

The PPY antibody (Novus #NB100-1793; RRID:AB_2268669) has been validated for use in FFPE tissue section staining (e.g., PMID: 35440614).

The ProGCG antibody (Cell Signaling #8233; RRID:AB_10859908) was generated by immunization with a synthetic peptide surrounding the Ala137 residue of human proglucagon (detailed immunogen information proprietary) and has been validated for use in FFPE tissue section staining (e.g., PMID: 30713109).

Additional validation information for all pancreatic endocrine hormone-specific antibodies except the CHGA-specific double clone can be fund at Biomed Resource Watch (https://scicrunch.org/ResourceWatch). In the present study, we first assessed and adapted protocols for brightfield staining of pancreatic FFPE tissue sections with individual antibodies, and then proceeded by empirical determination of adjustments for integration of individual antibody staining into the MICSSS pipeline.

Plants

Seed stocks

Not applicable.

Novel plant genotypes

Not applicable.

Authentication

Not applicable.
